# MAFG as a Redox‐Sensitive Node Linking Peripheral Immune Reprogramming to Cognitive Risk in the Sepsis‐to‐Alzheimer's Disease Continuum: Single‐Cell Dissection and Adoptive‐Transfer Validation

**DOI:** 10.1002/cns.71201

**Published:** 2026-10-13

**Authors:** Xiaoyu Xiang, Tingting Li, Zhongxue Feng, Jing Yang, Zhiwen Gong, Lietao Wang, Lijun Wang, Wei Zhang

**Affiliations:** ^1^ Institute of Critical Care Medicine, West China Hospital Sichuan University Chengdu Sichuan Province China; ^2^ Institute of Critical Care Medicine, State Key Laboratory of Biotherapy and Cancer Center, West China Hospital Sichuan University Chengdu Sichuan Province China

**Keywords:** Alzheimer's disease, MAFG, neuroinflammation, sepsis, single‐cell transcriptomics

## Abstract

**Background:**

Sepsis survivors face an elevated long‐term risk of Alzheimer's disease (AD), yet the peripheral pathways linking systemic immune activation to chronic neurodegeneration remain undefined. We mapped peripheral immune reprogramming across sepsis and AD and tested a candidate regulatory node.

**Methods:**

A single‐cell atlas of peripheral immunity was built by integrating scRNA‐seq from human sepsis and AD cohorts, using clustering, SCENIC regulatory‐network inference and an exploratory pseudotime ordering. The resulting signature was validated in two clinical cohorts (ROC and survival analysis). MAFG‐associated subpopulations and redox status were profiled in APP/PS1, cecal ligation and puncture (CLP) and composite models, and causality was tested by lentiviral MAFG knockdown and adoptive transfer into APP/PS1 recipients.

**Results:**

Peripheral immunity was myeloid‐skewed in sepsis and lymphoid‐dominated in AD, with APP‐expressing common myeloid progenitors (CMPs), dendritic cells (DCs), and platelets forming an APP‐enriched compartment. A core transcription‐factor network differed between disease states, yielding a four‐gene signature (MAFG, NFIL3, RGS10, FOXO3) with cross‐cohort diagnostic and prognostic value. MAFG was upregulated in septic patients and associated with poorer survival, subpopulation expansion, and systemic oxidative stress. Superimposed CLP aggravated amyloid deposition, microglial activation, and memory deficits in APP/PS1 mice, whereas MAFG silencing restored Aβ uptake and, on adoptive transfer, reduced amyloid burden, attenuated microgliosis, and preserved cognition.

**Conclusions:**

Peripheral immune states differ across the sepsis‐to‐AD spectrum and are captured by a MAFG‐centered signature. Independent of any trajectory assumption, loss‐of‐function and adoptive‐transfer experiments identify MAFG as a causal, redox‐sensitive node constraining peripheral Aβ clearance and a candidate biomarker and therapeutic target in sepsis survivors.

## Graphical abstract

Sepsis reprograms circulating immune cells, raising the redox‐sensitive transcription factor MAFG in myeloid progenitors, dendritic cells, and platelets and suppressing their amyloid‐β scavenger receptors. Silencing MAFG restores amyloid‐β clearance, reduces cerebral plaques and microglial activation, and preserves cognition in APP/PS1 mice.

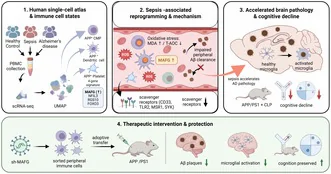

## 1 Introduction

Sepsis is a life‐threatening syndrome caused by a dysregulated host response to infection, and survivors frequently suffer from long‐term cognitive impairment. Epidemiological studies indicate that these individuals face a significantly elevated risk of developing Alzheimer's disease and related dementias (ADRD) [1]. Both clinical observations and animal models confirm that sepsis accelerates cognitive decline against a pre‐existing Alzheimer's pathological background, exacerbating amyloid‐β (Aβ) deposition and aberrant tau phosphorylation [2, 3, 4]. Sepsis‐associated encephalopathy (SAE) is a key intermediate phenotype in this transition, in which systemic inflammation disrupts the blood–brain barrier (BBB) and triggers neuroinflammation, oxidative stress, and synaptic dysfunction in critical regions such as the hippocampus [5, 6, 7, 8]. Mechanistically, sepsis also induces aberrant microglial activation and C1q‐mediated excessive synaptic pruning, impairing hippocampal plasticity [6] and shares with AD several core pathological cascades including mitochondrial dysfunction, ROS accumulation, neurotransmitter imbalance, and tau pathology progression [5, 9, 10, 11]. These overlapping cascades suggest that systemic, rather than purely central, dysregulation may be a feature shared by both conditions. Despite advances in supportive care, the molecular mechanisms bridging acute systemic inflammation and chronic neurodegeneration remain poorly understood, leaving sepsis survivors without effective biomarkers or targeted interventions for cognitive risk stratification.

The peripheral immune system has emerged as a critical link in this cross‐barrier pathology. The monocyte/macrophage lineage can act as a “peripheral sink” to facilitate efflux of brain‐derived Aβ into the circulation [12], and acute peripheral inflammation has been shown to enhance monocyte infiltration and reduce cerebral Aβ deposition in animal models [4]. Conversely, persistent immune activation and redox imbalance induced by sepsis can impair this clearance pathway [13], and additional gut‐brain axis disturbances—including microbiota dysbiosis, SCFA depletion, and NLRP6 inflammasome dysfunction—further exacerbate neuroinflammation [14, 15, 16, 17, 18, 19], positioning the peripheral immune compartment as a tractable but mechanistically underexplored link between these two conditions. However, peripheral immune cells are highly heterogeneous and undergo dynamic reprogramming during systemic inflammation, which masks cell‐type‐specific drivers in conventional bulk RNA‐seq data. Single‐cell RNA sequencing (scRNA‐seq) enables high‐resolution deconvolution of immune cell heterogeneity and dynamic reprogramming in sepsis, providing a framework for identifying key regulatory nodes [20, 21, 22, 23, 24, 25]. Yet a systematic single‐cell map that integrates sepsis and AD cohorts and dissects the regulatory networks that distinguish them has not been reported, and no peripheral biomarker has been validated for early risk stratification of sepsis‐associated cognitive impairment.

In this study, we integrated scRNA‐seq datasets from human sepsis and AD cohorts to construct a high‐resolution single‐cell atlas of peripheral immunity in both disease states. Using SCENIC‐based gene regulatory network analysis, together with an exploratory pseudotime ordering used only for hypothesis generation, we identified a core network of transcription factors whose inferred activity differs between early myeloid progenitors and terminally differentiated dendritic cells and platelets across disease states, and prioritized the redox‐sensitive transcription factor MAFG as a candidate hub associated with these differences. We further evaluated a MAFG‐centered four‐gene signature in dual clinical cohorts and characterized its associated immune subpopulations through flow cytometry and oxidative stress assays in composite APP/PS1 and CLP murine models. Moreover, we demonstrate that superimposing CLP‐induced sepsis on APP/PS1 mice aggravates amyloid burden, microglial activation, and recognition and spatial‐memory deficits, and that lentiviral silencing of MAFG in peripheral immune cells restores Aβ uptake while dampening pro‐inflammatory signaling; adoptive transfer of MAFG‐silenced cells into APP/PS1 recipients reduces cerebral amyloid burden, attenuates microglial reactivity, and preserves cognition. Collectively, these findings identify MAFG as a causal, targetable node linking peripheral immune reprogramming to Alzheimer‐like pathology, and a candidate biomarker and interventional target for the early stratification and management of sepsis‐related cognitive risk.

## 2 Material and Methods

Full protocols, reagents, antibodies, primers, and gating strategies are provided in the Supplementary Methods and Tables S1 and S2.

### 2.1 Datasets

Bulk RNA‐seq datasets were retrieved from GEO: GSE135838 (brain tissue; sepsis *n* = 12, controls *n* = 12; test set) [26], GSE65682 (PBMCs; sepsis *n* = 760, controls *n* = 42; validation set) [14, 27], and an in‐house PBMC cohort (sepsis *n* = 27, controls *n* = 59). Batch effects were corrected with “sva” and normalization performed with “limma” [28]. scRNA‐seq data of PBMCs from 71 individuals were integrated: 26 healthy controls (HC; GSE226602, GSE181279, GSE167363) [29, 30, 31], 31 ad patients (GSE226602, GSE181279) [29, 30] and 14 sepsis patients (GSE151263, GSE167363) [31, 32] (Table S1A). Donor age, sex, and 10× Genomics chemistry version for each contributing dataset are reported in Table S1B; donor age did not differ significantly across disease states (Kruskal–Wallis *p* = 0.456; Figure S9A).

### 2.2 Single‐Cell Processing, Composition and Differential Expression

Matrices were processed in Seurat [33] with standard quality control, normalization, Harmony batch correction, PCA/UMAP and unsupervised clustering, with clusters annotated by canonical markers (Supplementary Methods). Differentially expressed genes (DEGs) were identified by Wilcoxon rank‐sum test with Bonferroni correction (|log2FC| > 1, FDR < 0.05) and key disease‐state comparisons were confirmed by patient‐level pseudo‐bulk analysis to limit pseudo‐replication. Disease preference was quantified by the STARTRAC‐dist index, the ratio of observed to expected cell numbers (Ro/e) and odds ratios (OR), with OR > 1.5 or Ro/e > 1 indicating enrichment [34].

### 2.3 Trajectory Inference and Regulatory‐Network Analysis

Pseudotime trajectories were constructed with Monocle2 [35]. Because the integrated data derive from independent, cross‐sectional sepsis and AD individuals rather than a longitudinally sampled cohort, the resulting ordering cannot represent an authentic temporal or pathophysiological progression; it was used only as an exploratory, hypothesis‐generating description, and no primary conclusion depends on it (see Section 4). Regulons were inferred with SCENIC [36]: GENIE3 co‐expression modules were refined by RcisTarget motif enrichment (normalized enrichment score > 3.0) and scored per cell by AUCell, with highly variable regulons defined by activity variance across disease states.

### 2.4 Age‐ and Sex‐Adjusted Differential Expression

To assess whether donor age confounded the disease‐state comparisons, the core differential‐expression analyses were repeated with age and sex as covariates using two models: a cell‐level logistic‐regression model (Seurat FindMarkers, test.use = “LR”, latent.vars = age + sex) and a donor‐level pseudo‐bulk model (DESeq2, design ~ age + sex + disease state). The two models were compared by genome‐wide log2 fold‐change correlation, by signature‐gene effect sizes, and in an age‐matched sub‐cohort. Age equivalence across disease states was tested by two one‐sided tests. Full details are given in the Supplementary Methods.

### 2.5 Enrichment and Diagnostic Evaluation

DEGs were annotated with “clusterProfiler” (GO and KEGG; *q* < 0.01, *p* < 0.05). Pathway dysregulation was assessed by GSEA against MSigDB [37] and by single‐sample GSEA (GSVA) in the peripheral‐blood (GSE65682) and brain‐tissue (GSE135838) cohorts. Diagnostic and prognostic performance was evaluated by ROC analysis with area under the curve (AUC) [38].

### 2.6 Clinical Validation Cohorts

Two independent in‐house cohorts were used: a primary PBMC sequencing cohort (29 controls, 59 sepsis patients with admission Glasgow Coma Scale [GCS] scores; Genome Sequence Archive HRA002988) [39] and a secondary serum cohort (9 controls, 15 sepsis patients). Blood was collected within 24 h of admission.

### 2.7 Animal Models

Experiments were approved by the Animal Welfare Committee of West China Hospital, Sichuan University (No. 2021‐S‐012) and followed NIH guidelines. Male C57BL/6 and age‐matched male APP/PS1 double‐transgenic mice (B6‐Tg(PrP‐hAPP/hPS1); Beijing HFK Bio‐Technology) were housed under specific‐pathogen‐free conditions. Polymicrobial sepsis was induced by cecal ligation and puncture (CLP) under isoflurane anesthesia [40]; sham mice underwent laparotomy without ligation or puncture. Four groups were studied: Sham, CLP, APP/PS1 and the composite APP/PS1 + CLP model. Blood and bone marrow were harvested at pre‐defined time points (8 h–30 days) for molecular and flow‐cytometric profiling. Behavioral phenotyping was performed 30 days after surgery, in the post‐acute recovery phase (Section 2.9); moribund animals meeting humane end‐points were excluded before testing.

### 2.8 Molecular and Cellular Assays

Western blotting and RT‐qPCR (2^−ΔΔCt^; 18S rRNA reference) were performed on PBMCs. Multicolor flow cytometry of murine blood and bone marrow quantified common myeloid progenitors (CMPs), CD11c‐enriched dendritic cells (DCs), and platelets, with intracellular MAFG detected using a Foxp3/transcription‐factor staining buffer set (Supplementary Methods). Serum malondialdehyde (MDA) and total antioxidant capacity (T‐AOC) were measured with commercial colorimetric kits.

### 2.9 Behavioral Testing

Behavioral testing in the composite APP/PS1 + CLP model began in the post‐acute recovery phase (30 days after CLP; timeline in Figure S5C), after confirmation that body weight, daily food intake, murine sepsis score, and home‐cage activity no longer differed from sham‐operated controls (Figure S10). Cognition and locomotion were assessed by a blinded investigator with automated video tracking, using identical procedures throughout the study: novel object recognition (recognition and discrimination indices, with distance and velocity as locomotor controls) and Y‐maze (spontaneous alternation and novel‐arm variant) in the composite model, with the Morris water maze (acquisition, probe trial, and occupancy heatmaps) additionally performed in the adoptive‐transfer cohort. Locomotor and body‐weight parameters recorded at the time of testing were used to exclude residual sickness behavior as a confounder. Full parameters are given in the Supplementary Methods.

### 2.10 Neuropathology

Perfusion‐fixed brains were cryosectioned; amyloid plaques were detected by 4G8 immunostaining and Methoxy‐X04 histochemistry, and microglia by Iba1 immunofluorescence (Supplementary Methods). Amyloid burden (plaque load, density, count and mean area) and Iba1^+^ parameters were quantified in cortex and hippocampus in ImageJ/FIJI by a blinded investigator; microglial morphology was reconstructed by skeletonization (AnalyzeSkeleton) and Sholl analysis. Brain Aβ1–42 was measured by sandwich ELISA.

### 2.11 MAFG Knockdown, Functional Profiling and Adoptive Transfer

Peripheral immune cells were isolated by density‐gradient centrifugation and fluorescence‐activated cell sorting, then transduced with lentivirus encoding MAFG‐targeting (sh‐MAFG) or non‐targeting (sh‐NC) shRNA; knockdown was confirmed by RT‐qPCR and intracellular MAFG flow cytometry. Aβ internalization was quantified by flow cytometry after trypan‐blue quenching of non‐internalized fluorescence. Cytokine secretion (IL‐1β, IL‐6, TNF‐α) was measured by ELISA with or without lipopolysaccharide, and scavenger‐receptor and pro‐inflammatory transcripts by RT‐qPCR (Supplementary Methods). For adoptive transfer, 6‐month‐old APP/PS1 recipients received four serial tail‐vein transfers of sh‐NC or sh‐MAFG cells (timeline in Figure 6F); knockdown persistence was verified in recipient blood before behavioral and neuropathological analysis.

### 2.12 Statistical Analysis

Analyses used R and GraphPad Prism. Data are mean ± SEM. Two groups were compared by unpaired Student's *t*‐test and multiple groups by one‐ or two‐way ANOVA with Tukey's post hoc test; categorical variables by Chi‐squared test; survival by Kaplan–Meier analysis with log‐rank test. Two‐sided *p* < 0.05 was considered significant.

## 3 Results

### 3.1 A Single‐Cell Atlas Defines an APP‐Expressing Peripheral Axis of CMPs, DCs, and Platelets

To delineate the peripheral immune landscape in sepsis and AD, scRNA‐seq data from 71 individuals (26 HC, 31 ad, 14 sepsis) were integrated through a unified pipeline (Figure 1A). After quality control, 379,666 cells and 48,068 genes were retained (Table S3). Unsupervised clustering resolved 28 clusters grouped into 9 lineages by canonical markers (Figure 1B; Figure S1A,B; Table S4). Sepsis showed expansion of monocyte/macrophage, platelet and B‐cell compartments, whereas HC and AD landscapes were lymphoid‐dominated (Figure S1C,F–G). Preference modeling confirmed enrichment of platelets, HSCs and Mon/Mac in sepsis versus T, NK and T/NKT cells in HC and AD, at the lineage level (OR and Ro/e; Figure 1C,D) and at the cluster level (Figure S1D,E).

**FIGURE 1:**
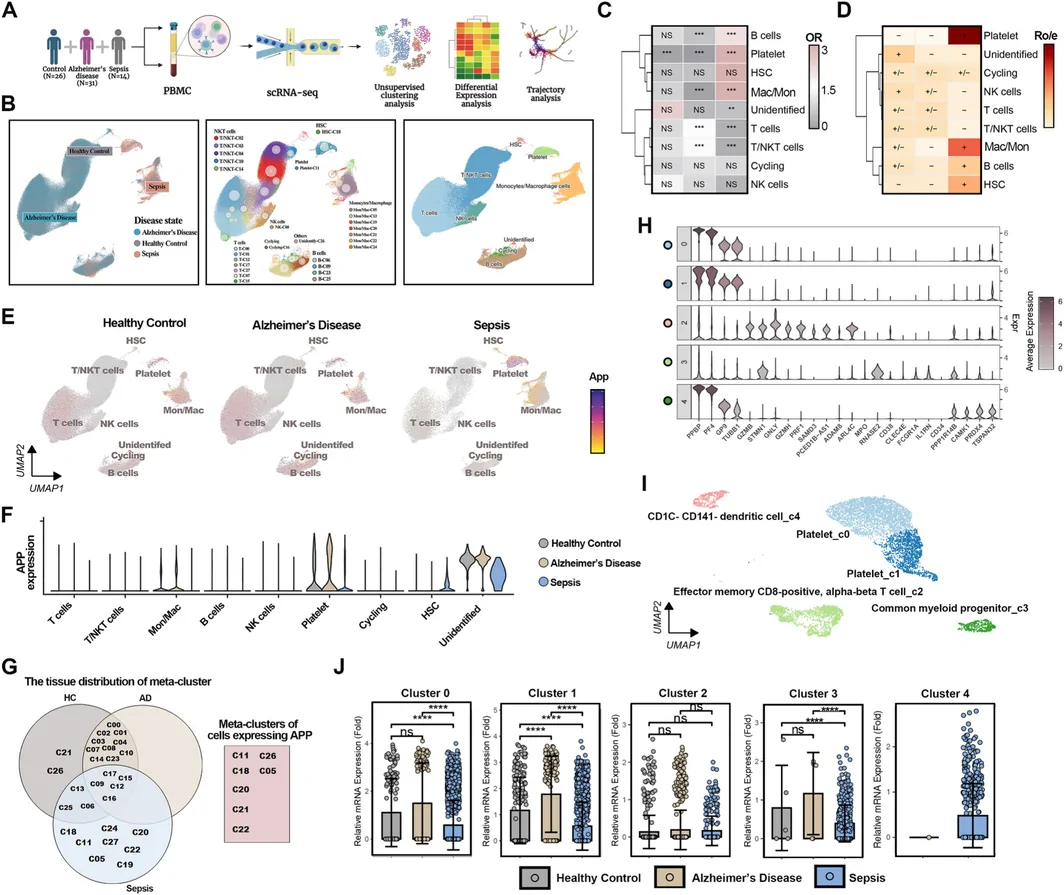
Single‐cell transcriptomic atlas of peripheral immunity and APP‐restricted meta‐cluster identification across sepsis and AD cohorts. (A) Schematic of the integrated scRNA‐seq analytical pipeline, showing PBMC sample collection from HC (*n* = 26), AD (*n* = 31), and Sepsis (*n* = 14) cohorts and the downstream computational framework, including unsupervised clustering, differential expression, trajectory, and cell–cell communication analyses. (B) UMAP projections of integrated PBMC datasets, colored by disease state (left), the 28 unsupervised clusters (middle), and the 9 annotated major immune lineages (right). (C, D) Tissue/disease preference modeling at the lineage level. Heatmaps show the odds ratios (OR; C) and the ratio of observed to expected cell numbers (Ro/e; D). An OR > 1.5 indicates preference for the corresponding disease state; symbols in D indicate enrichment preference (+, positive; ±, neutral; −, negative). (E) Split UMAP feature plots showing the transcriptional localization of APP in HC, AD, and Sepsis cohorts. Color scale indicates normalized expression. (F) Violin plot showing APP expression across the nine major immune lineages, stratified by disease state. (G) Venn diagram showing the overlap and disease‐preferential enrichment of cell clusters across HC, AD, and Sepsis cohorts. Summary list of the APP‐expressing meta‐clusters (C05, C11, C18, C20, C21, C22, C26) identified by intersecting disease‐preferential and APP^+^ subpopulations. (H) Violin plots showing the expression of canonical markers across the five terminal subpopulations re‐clustered from the APP‐enriched subtypes (Clusters 0, 2, 7, and 9). (I) UMAP embedding of the five terminal subpopulations: Platelets (Clusters 0 and 1), effector memory CD8^+^ αβ T cells (Cluster 2), common myeloid progenitors (CMPs; Cluster 3), and CD1C^−^CD141^−^ dendritic cells (Cluster 4). (J) Box‐and‐scatter plots comparing APP expression across HC, AD, and Sepsis cohorts in the five terminal subpopulations. Significant differences are observed in platelets (Clusters 0, 1), CMPs (Cluster 3), and CD1C^−^CD141^−^ DCs (Cluster 4) (****p < 0.0001), but not in effector memory CD8^+^ T cells (Cluster 2). ns, not significant.

Mapping APP showed a restricted expression pattern, strongest in platelets, monocyte/macrophage subpopulations and a small unidentified subset (Figure 1E,F), and cluster‐level enrichment in C11, C20–C22, C26, and C05 with disease‐dependent intensity (Figure S1H). Intersecting disease‐preferential clusters with APP^+^ subpopulations defined seven meta‐clusters shared by both disease states (C05, C11, C18, C20–C22, C26; Figure 1G).

Sub‐clustering of these meta‐clusters resolved 14 functional subtypes spanning neutrophil, macrophage, monocyte, DC, megakaryocyte, NK, B and T identities (Figures S2A–C and S3C,D; Tables S5 and S6), with expansion of neutrophil and monocyte subsets in sepsis and of macrophage and DC_c9‐FCER1A subsets in HC and AD (Figure S2D–F). Within these subtypes, APP was most enriched in Cluster 9 in AD (Figure S3A,B). Re‐clustering the APP‐enriched subtypes identified five terminal subpopulations: platelets, effector memory CD8^+^ αβ T cells, CMPs and CD1C^−^CD141^−^ DCs (Figure 1H,I; defining DEGs and additional canonical markers in Figure S3E,F). APP expression differed across cohorts in platelets, CMPs and DCs (*p* < 0.0001) but not in CD8^+^ T cells (Figure 1J), identifying CMPs, DCs and platelets as the principal peripheral APP‐expressing populations. APP transcription alone does not establish pathological Aβ production; these populations are therefore interpreted as transcriptionally APP‐enriched (see Section 4).

### 3.2 Exploratory Pseudotemporal Modeling Suggests Epigenetic and AD‐Risk Gene Differences Between Cell States

Exploratory trajectory inference rooted in CMPs (predominantly sepsis‐derived) bifurcated toward two AD‐dominated terminal states, DCs and platelets, with a separate HC branch (Figure S4A–C); this ordering is cross‐sectional and does not represent progression over time. Transcriptional dynamics resolved into four co‐expression modules that converged on neurodegenerative and neurotrophin signaling pathways (Figure S4D; Table S7). Established AD‐risk genes [41] were expressed predominantly in sepsis‐derived CMPs and, by contrast, in AD‐derived terminal DCs (Figure S4E; Table S8), and epigenetic modulators [42] were lower in sepsis‐derived DCs yet higher in AD‐derived DCs and platelets (Figure S4F). Because this ordering is cross‐sectional, these differences are treated as hypothesis‐generating and tested directly below and in the mouse experiments.

### 3.3 SCENIC Identifies a Core Transcription‐Factor Network Differing Between Disease States

Pseudo‐bulk differential expression identified 122 genes upregulated in sepsis‐derived CMPs and 59 and 144 genes enriched in AD‐derived DCs and platelets, respectively (Figure 2A–D; Table S9). SCENIC identified 197 highly variable regulons (Figure 2E; Table S10). Integrating 779 state‐associated and disease‐specific DEGs (Figure 2F) with these regulons yielded a core network of 13 regulators, including MAFG, NFIL3, and FOXO3 (Figure 2G; Table S10). Regulon activity was highest in sepsis‐derived CMPs and, separately, in AD‐derived terminal DCs and platelets (Figure 2H,I), a between‐state difference rather than a temporal reassignment. Because peripheral mononuclear phagocytes mediate systemic Aβ clearance [12], canonical scavenger receptors were also examined: TLR2, CD33, MSR1, and SYK were downregulated in sepsis‐derived CMPs but restored in AD‐derived DCs (Figure 2J), with the 13 core genes enriched in the AD landscape (Figure 2K). SCENIC infers putative regulatory associations from co‐expression and motif enrichment rather than direct causal regulation; these data therefore describe a pattern that may relate to altered peripheral Aβ clearance.

**FIGURE 2:**
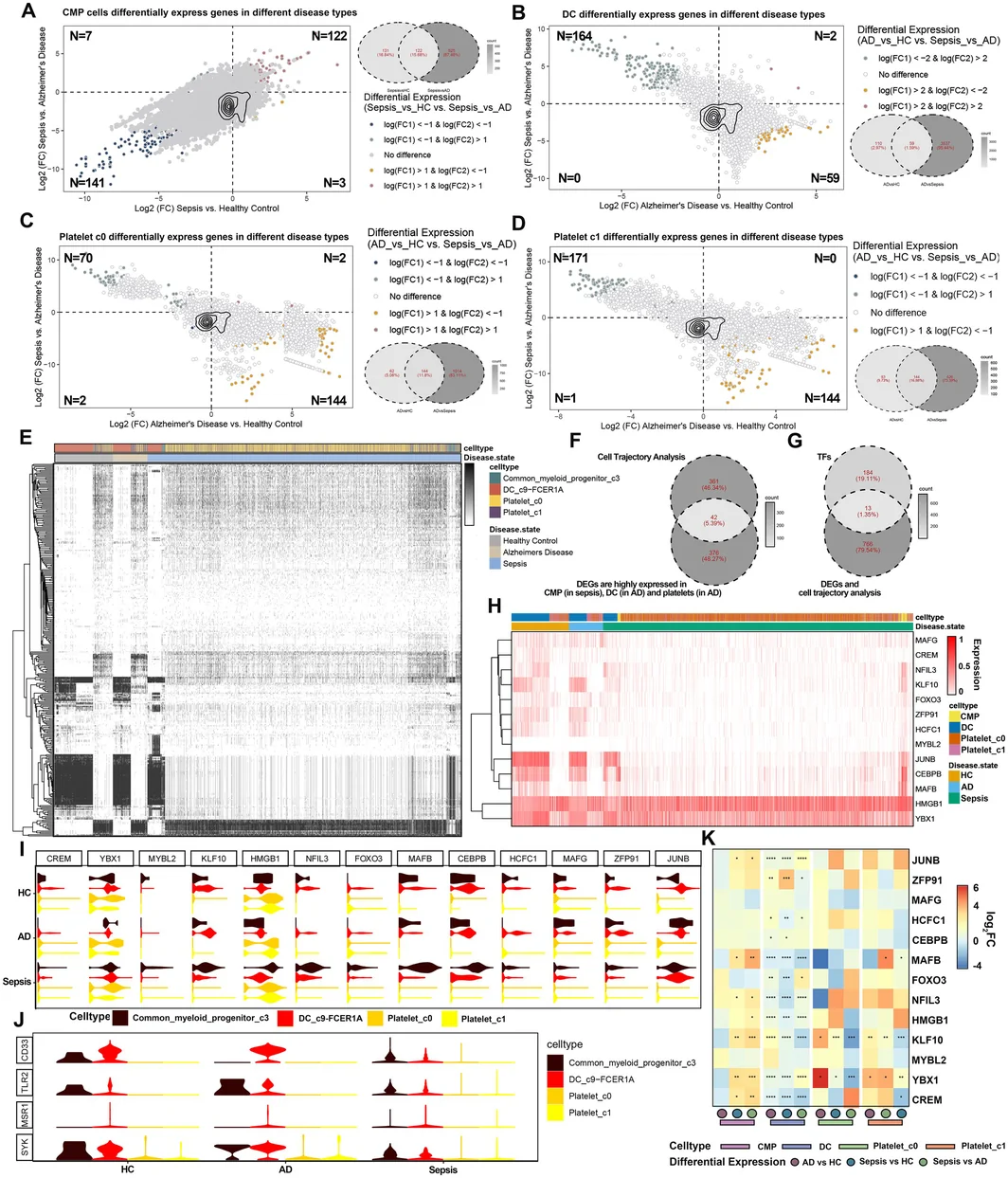
SCENIC analysis identifies a core transcription factor network differing between sepsis and AD. (A–D) Cross‐shaped volcano plots and Venn diagrams showing DEGs upregulated in Sepsis‐derived CMPs (A), AD‐derived CD1C^−^CD141^−^ DCs (B), and AD‐derived Platelet_c0 (C) and Platelet_c1 (D) subpopulations. (E) Heatmap showing the AUCell scores of 197 highly variable regulons identified by SCENIC. (F, G) Stepwise intersection strategy for identifying core regulatory hubs. Venn diagrams show the integration of 779 state‐associated and disease‐specific DEGs (F) with the 197 regulons, yielding 13 core transcription factors (G). (H, I) Expression of the 13 core transcription factors across cell states and disease groups. Heatmap (H) and grouped violin plots (I) show predominant expression in sepsis‐derived CMPs and, separately, in AD‐derived terminal DCs and platelets. (J) Box plots showing the expression of canonical Aβ uptake receptors (TLR2, CD33, MSR1, SYK), with lower expression in sepsis‐derived CMPs and higher expression in AD‐derived DCs. (K) Heatmap showing the logFC and statistical significance (−log_10_[*p*‐value]) of the 13 core transcription factors.

### 3.4 Two‐Cohort Clinical Screening Identifies a Core Four‐Gene Signature

GO and KEGG annotation of the 13 regulators highlighted myeloid, dendritic, and lymphocyte differentiation and TNF and IL‐17 signaling (Figure 3A; Table S7). Comparison with 29 established AD blood biomarkers [43] revealed substantial overlap in the AD PBMC cohort, with upregulation of GUSB, PER1, and UBE2I in sepsis‐derived DCs and platelets (Figure 3B,C; Table S8).

**FIGURE 3:**
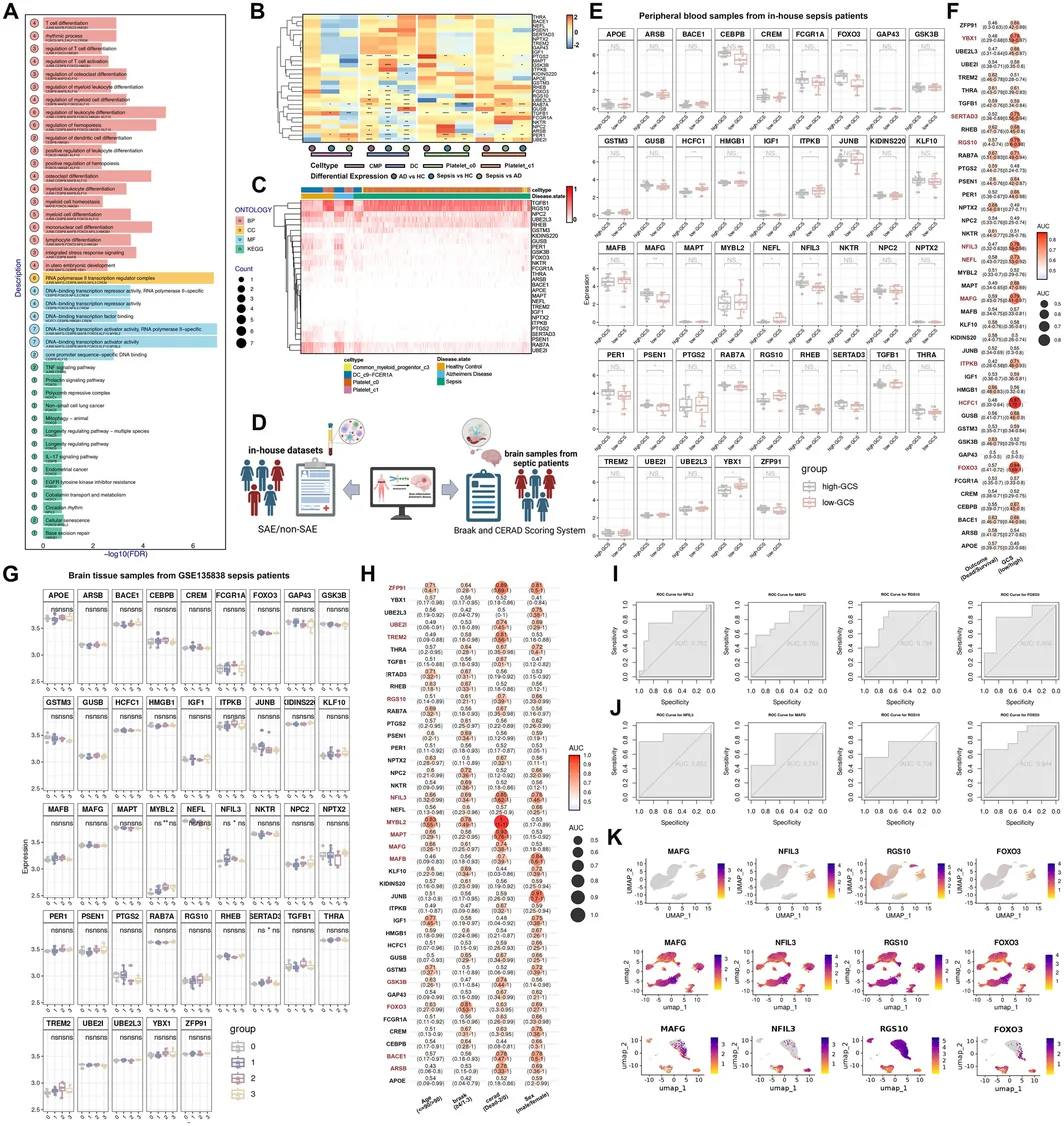
Functional profiling and clinical screening identify a core diagnostic signature. (A) GO and KEGG enrichment of the 13 core transcription factors. (B, C) Expression of 29 established AD blood biomarkers. Heatmap of statistical significance (B) and scaled expression (C) across HC, AD, and Sepsis PBMCs. (D) Schematic of the two‐cohort clinical validation strategy using peripheral GCS scores and central CERAD neuropathological scores. (E, F) Diagnostic evaluation in the peripheral blood cohort. Box plots (E) and AUC heatmap (F) show the predictive value of candidate genes for cognitive impairment severity stratified by GCS. (G, H) Diagnostic evaluation in the brain tissue cohort (GSE135838). Box plots (G) and AUC heatmap (H) show the association of candidate genes with AD‐like pathology stratified by CERAD score. (I, J) ROC curves of the four‐gene signature (MAFG, NFIL3, RGS10, FOXO3) in the peripheral blood (I) and brain tissue (J) cohorts (AUC > 0.7). (K) UMAP feature plots showing the spatial enrichment of the four candidate biomarkers within the APP^+^ immune populations.

A two‐cohort validation strategy was then applied (Figure 3D). In the in‐house sepsis cohort stratified by GCS score (Tables S11 and S12), ROC analysis identified 10 genes, including MAFG, NFIL3, and FOXO3, with AUC > 0.7 for neurological impairment during acute sepsis (Figure 3E,F; Table S13). In the GSE135838 brain‐tissue cohort stratified by CERAD score (Table S14), 13 genes were associated with AD‐like neuropathology (AUC > 0.7) (Figure 3G,H). Integrating both panels yielded a consensus four‐gene signature (MAFG, NFIL3, RGS10, FOXO3) validated in blood (Figure 3I) and brain tissue (Figure 3J) with AUC > 0.7 and enriched within APP^+^ immune populations (Figure 3K).

### 3.5 Prognostic Evaluation and In Vivo Validation Identify MAFG as a Core Pathogenic Driver

Kaplan–Meier analysis in a large sepsis cohort (GSE65682) showed significant prognostic stratification for FOXO3 and MAFG (*p* < 0.05; Figure 4A). ssGSEA revealed that FOXO3 was primarily associated with neuronal signaling, whereas MAFG was enriched in both compartments: neutrophil degranulation, platelet activation, and intracellular trafficking in blood (Figure 4B), and calcium signaling, neurotransmitter regulation, and GPCR pathways in brain tissue (Figure 4C). MAFG was prioritized for its combination of prognostic stratification (Figure 4A), the highest cross‐cohort diagnostic AUC (Figure 3F–H), consistent peripheral and central pathway enrichment (Figure 4B,C), and its established role as a redox‐sensitive transcription factor [44, 45, 46]. RT‐qPCR and Western blotting confirmed MAFG upregulation in peripheral blood of CLP and APP/PS1 mice (Figure 4D,E; uncropped blots in Figure S5A; Table S15).

**FIGURE 4:**
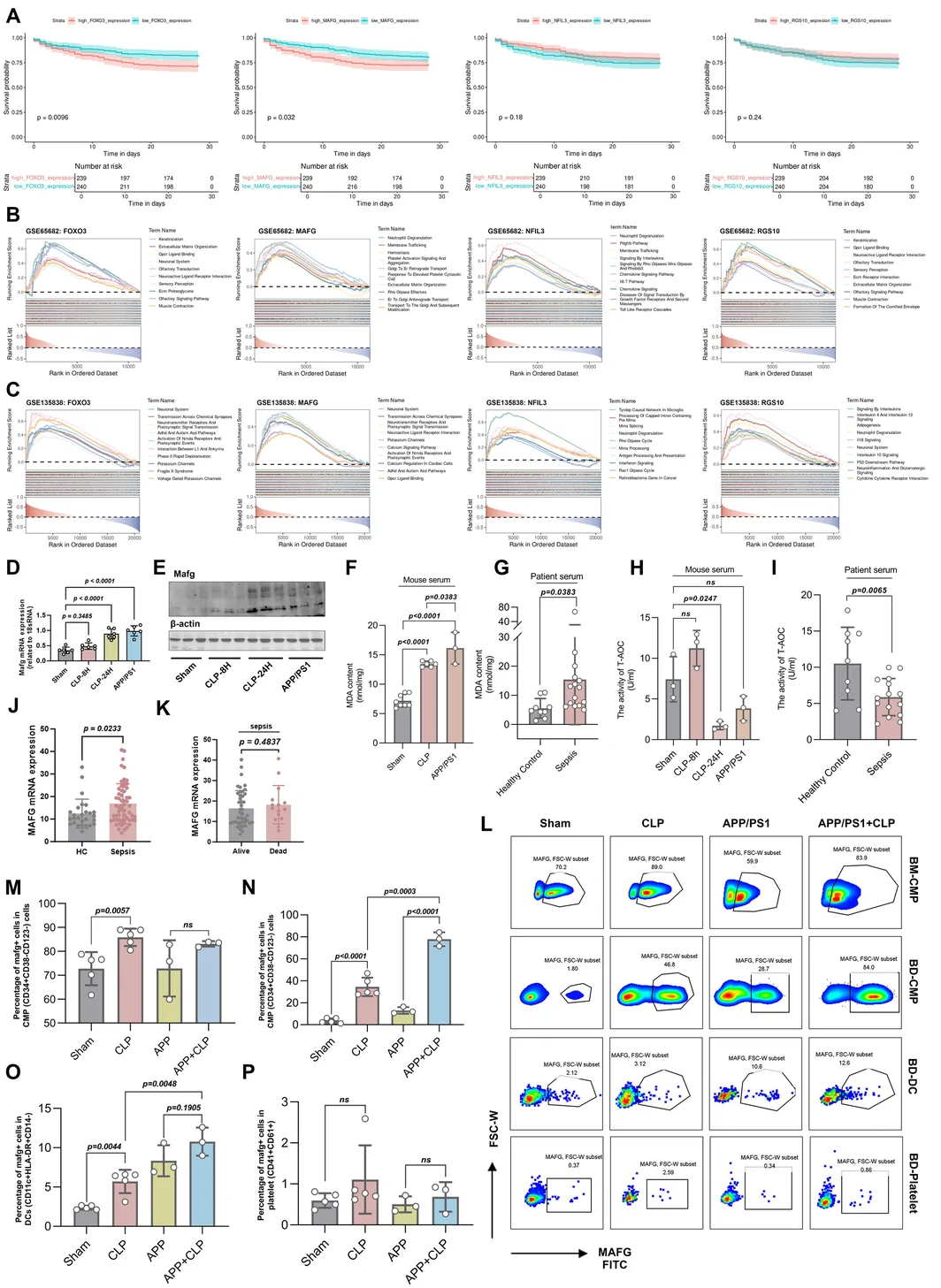
MAFG as a prognostic biomarker associated with peripheral immune differences and systemic redox imbalance. (A) Kaplan–Meier survival analysis of sepsis patients (GSE65682) stratified by FOXO3, MAFG, NFIL3, and RGS10 expression; FOXO3 and MAFG showed significant prognostic stratification (*p* < 0.05). (B, C) Single‐sample GSEA (ssGSEA) functional profiling of the four candidate biomarkers in the peripheral blood (B; GSE65682) and brain tissue (C; GSE135838) cohorts. (D) RT‐qPCR showing Mafg mRNA upregulation in the peripheral blood of Sham, CLP, and APP/PS1 mice. (E) Representative Western blot showing MAFG protein upregulation in the peripheral blood of Sham, CLP (8 and 24 h), and APP/PS1 mice, with β‐actin as loading control. (F, G) Serum malondialdehyde (MDA) levels in mouse models (F) and human sepsis patients (G). (H, I) Serum total antioxidant capacity (T‐AOC) in mouse models (H) and human sepsis patients (I). (J, K) MAFG mRNA expression in the peripheral blood of sepsis patients versus healthy controls (J) and in survivors versus non‐survivors (K). (L) Representative flow cytometry plots showing MAFG^+^ subsets in bone marrow CMPs (BM‐CMP), peripheral blood CMPs (BD‐CMP), DCs (BD‐DC), and platelets (BD‐Platelet) across Sham, CLP, APP/PS1, and APP/PS1 + CLP models. (M–P) Quantification of the percentage of MAFG^+^ cells in bone marrow CMPs (M), peripheral CMPs (N), DCs (O), and platelets (P). Data are presented as mean ± SEM. Statistical significance was assessed by unpaired Student's *t*‐test or ANOVA. ns, not significant.

Systemic oxidative stress paralleled these changes: serum MDA was elevated in murine models (Figure 4F) and human sepsis patients (Figure 4G), whereas T‐AOC showed an early transient rise after CLP followed by depletion at 24 h, mirroring findings in AD mice and patients (Figure 4H,I; Table S16). In the in‐house cohort, MAFG transcription was elevated in sepsis patients versus controls (Figure 4J) and higher in non‐survivors, indicating a trend toward increased mortality risk (Figure 4K). Multicolor flow cytometry showed that CLP expanded MAFG^+^ CMPs in bone marrow and blood, APP/PS1 mice accumulated MAFG^+^ DCs and platelets, and the composite model exhibited systemic expansion across all three subpopulations (Figure 4L–P; gating in Figure S5B; Table S17), consistent with the between‐state difference in MAFG activity described above. These data reflect peripheral immune and redox remodeling and do not, on their own, establish a contribution to central neurodegeneration.

### 3.6 Superimposed Sepsis Exacerbates Cognitive Decline and Central Neuropathology in APP/PS1 Mice

We next compared the composite APP/PS1 + CLP model with APP/PS1 controls to determine whether this peripheral remodeling is accompanied by aggravated central pathology.

Total distance moved and mean velocity were comparable between groups, excluding motor confounds (Figure 5A,B). Despite equivalent locomotion, APP/PS1 + CLP mice showed reduced discrimination and recognition indices (Figure 5C,D; *p* = 0.0156 and *p* = 0.0157, respectively) and diminished preferential exploration of the novel object on occupancy heatmaps (Figure 5E; Table S18). This deficit was reproduced in an independent validation cohort (Figure S5D–H; discrimination index, *p* = 0.0002; recognition index, *p* = 0.0005). In the Y‐maze (Figure 5J), composite mice spent less time in the novel arm (Figure 5F; *p* = 0.0087), showed lower spontaneous alternation (Figure 5G; *p* = 0.0029), and reduced novel‐arm preference on arm‐wise analysis (Figure 5H; Arm B, *p* = 0.0088; Arm C, *p* = 0.0011), with unchanged distance and velocity (Figure 5I).

**FIGURE 5:**
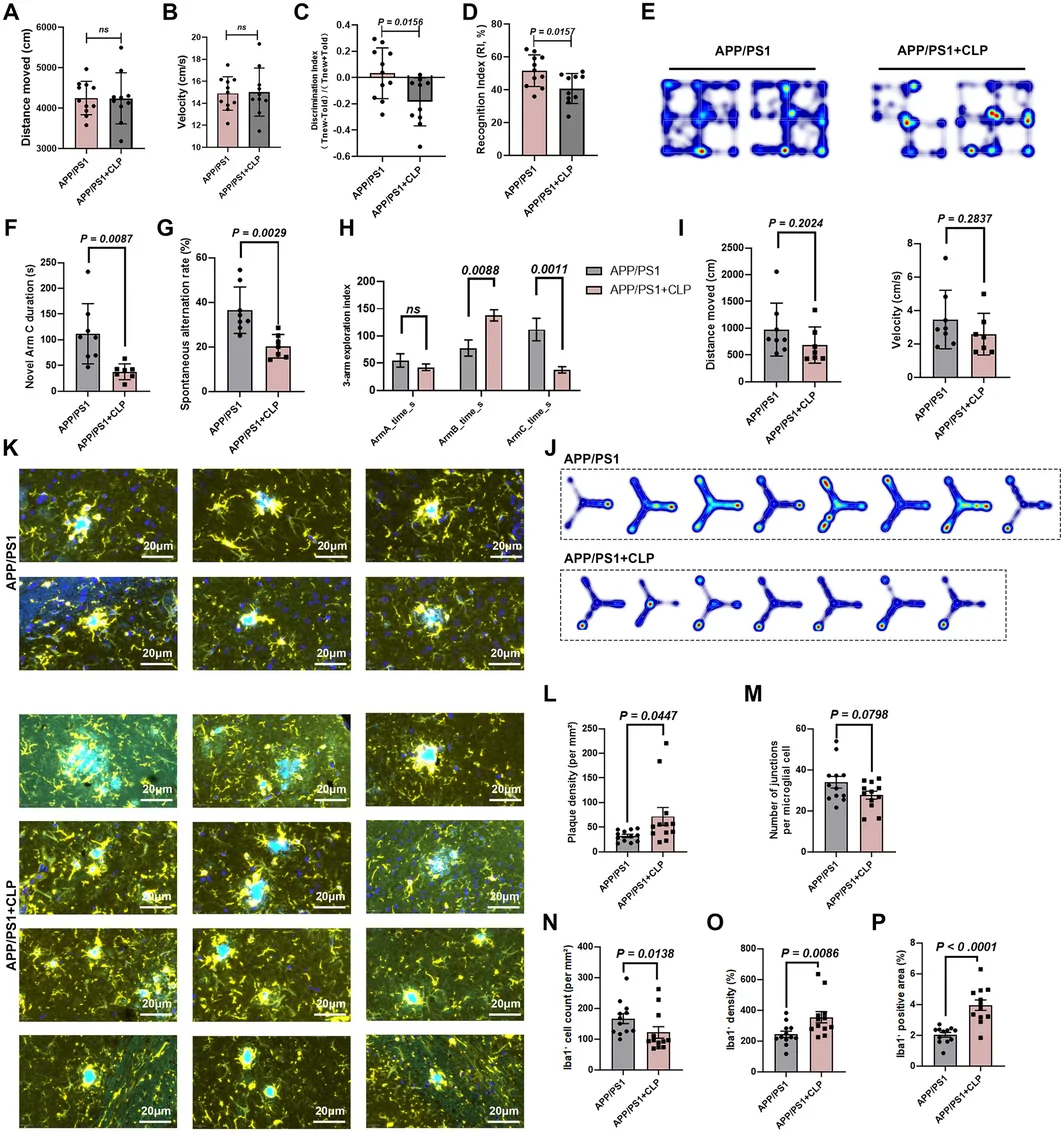
Superimposed sepsis exacerbates cognitive impairment and central neuropathology in APP/PS1 mice. Behavioral and neuropathological comparison of APP/PS1 and composite APP/PS1 + CLP mice. (A, B) Total distance moved (A) and mean velocity (B) in the recognition memory test, showing comparable locomotor activity between groups (NS). (C) Discrimination index [(Tnovel − Told)/(Tnovel + Told)] in the recognition memory test (*p* = 0.0156). (D) Recognition index (RI, %) in the recognition memory test (*p* = 0.0157). (E) Representative occupancy heatmaps of object exploration for APP/PS1 and APP/PS1 + CLP mice. (F) Time spent in the novel arm of the Y‐maze (*p* = 0.0087). (G) Spontaneous alternation rate in the Y‐maze (*p* = 0.0029). (H) Arm‐wise exploration time in the Y‐maze (Arm A, NS; Arm B, *p* = 0.0088; Arm C, *p* = 0.0011). (I) Distance moved and velocity during the Y‐maze test, showing no locomotor difference (*p* = 0.2034 and *p* = 0.2837, respectively). (J) Representative Y‐maze occupancy heatmaps (APP/PS1 vs. APP/PS1 + CLP). (K) Representative immunohistochemical images of amyloid plaques (blue) and Iba1^+^ microglia (yellow); scale bar, 20 μm. (L) Quantification of amyloid plaque density (per mm^2^; *p* = 0.0447). (M) Number of junctions per microglial cell (*p* = 0.0798). (N) Iba1^+^ cell count (*p* = 0.0138). (O) Iba1 staining density (*p* = 0.0086). (P) Iba1^+^ area fraction (*p* < 0.0001). Data are presented as mean ± SEM; statistical significance was assessed by unpaired Student's *t*‐test. NS, not significant.

At the time of testing—30 days after CLP—body weight, food intake, murine sepsis score and home‐cage activity had returned to the sham range and no longer differed between APP/PS1‐CLP and APP/PS1‐Sham mice (Figure S10), indicating that the observed deficits reflect persistent cognitive impairment in recovered animals rather than acute sickness behavior.

Brain sections co‐stained for amyloid plaques and Iba1 showed denser plaque deposition and clustering of reactive microglia around plaques in composite mice (Figure 5K; Figure S6A). Quantification confirmed increased plaque density (Figure 5L; *p* = 0.0447), a trend toward fewer junctions per microglial cell consistent with process retraction (Figure 5M; *p* = 0.0798), and increased Iba1^+^ cell counts (Figure 5N; *p* = 0.0138), staining density (Figure 5O; *p* = 0.0086) and area fraction (Figure 5P; *p* < 0.0001; Table S19). Complementary morphometric measures did not differ significantly (Figure S6B–G), indicating that aggravation was most pronounced in plaque density and Iba1 burden. These experiments establish an association between peripheral sepsis and aggravated central pathology but do not isolate MAFG as the mediator, nor do they establish that the behavioral deficits are AD‐specific rather than also reflecting post‐sepsis impairment.

### 3.7 MAFG Knockdown Restores Aβ Clearance, and Adoptive Transfer Attenuates Neuropathology and Cognitive Decline

To test whether MAFG is causal, it was silenced in sorted peripheral immune cells and the consequences of adoptive transfer were evaluated (Figure 6A).

**FIGURE 6:**
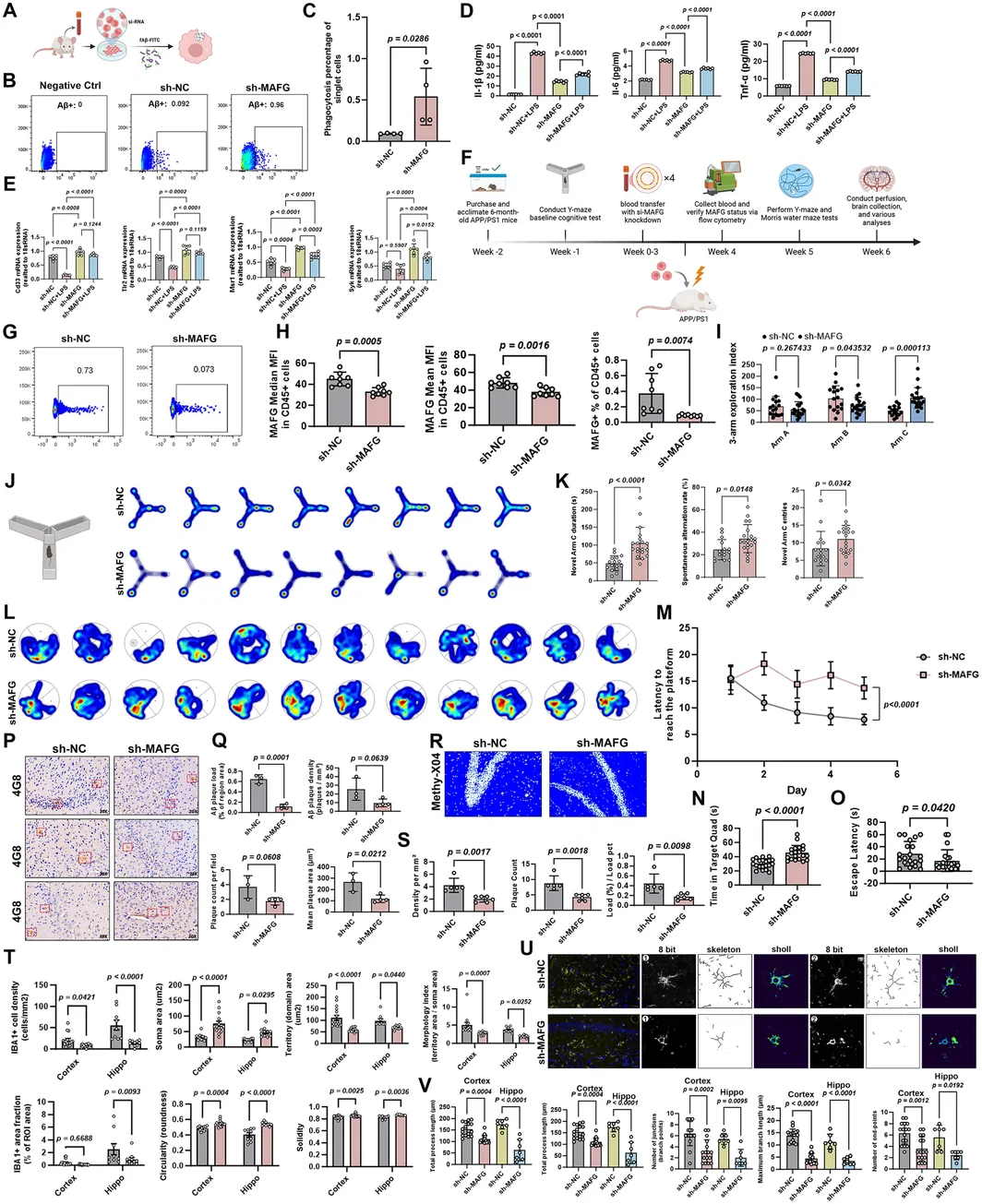
MAFG knockdown restores peripheral Aβ clearance, and adoptive transfer of MAFG‐silenced cells attenuates neuropathology and cognitive decline in APP/PS1 recipients. (A) Schematic of the in vitro MAFG‐knockdown workflow in sorted peripheral immune cells. (B) Representative flow cytometry of Aβ uptake (Aβ^+^) in Negative Ctrl, sh‐NC, and sh‐MAFG cells. (C) Quantification of the Aβ^+^ phagocytic percentage (sh‐NC vs. sh‐MAFG; *p* = 0.0286). (D) ELISA of pro‐inflammatory cytokines IL‐1β, IL‐6, and TNF‐α in sh‐NC versus sh‐MAFG cells. (E) RT‐qPCR of Aβ‐clearance/scavenger receptor genes (top) and pro‐inflammatory genes (bottom) in sh‐NC versus sh‐MAFG cells. (F) Schematic and timeline of the adoptive‐transfer experiment: 6‐month‐old APP/PS1 recipients received serial transfers of control (sh‐NC) or MAFG‐silenced (sh‐MAFG) peripheral cells, followed by Y‐maze and Morris water maze testing and brain collection. (G) Representative flow cytometry verifying MAFG knockdown in the transferred compartment. (H) Quantification of MAFG median MFI (*p* = 0.0005), mean MFI in CD45^+^ cells (*p* = 0.0016), and MAFG^+^ percentage of CD45^+^ cells (*p* = 0.0074). (I) Y‐maze 3‐arm exploration index in recipients (Arm A, NS; Arm B, *p* = 0.043152; Arm C, *p* = 0.000113). (J) Representative Y‐maze occupancy heatmaps. (K) Y‐maze metrics: Novel‐arm duration, spontaneous alternation rate, and novel‐arm entries (e.g., *p* = 0.0342). (L) Representative Morris water maze occupancy heatmaps. (M) Escape latency across training days (*p* < 0.0001). (N) Time in the target quadrant (*p* = 0.0001). (O) Probe‐trial escape latency (*p* = 0.0420). (P) Representative 4G8 amyloid immunostaining of recipient brains. (Q) Quantification of 4G8 amyloid pathology: Aβ plaque load (*p* = 0.0001), plaque density (*p* = 0.0639), plaque count per field (*p* = 0.0608), and mean plaque area (*p* = 0.0212). (R) Representative Methoxy‐X04 amyloid staining with DAPI nuclear counterstain. (S) Quantification of Methoxy‐X04 pathology: Plaque density (*p* = 0.0017), plaque count (*p* = 0.0018), and amyloid load (*p* = 0.0098). (T) IBA1^+^ microglial quantification in cortex and hippocampus: Cell density, area fraction, soma area, territory (domain) area, morphology index (territory area/soma area), circularity, and solidity. (U) Representative IBA1^+^ microglial morphology showing 8‐bit, skeleton, and Sholl reconstructions (sh‐NC vs. sh‐MAFG; cortex and hippocampus). (V) Skeleton/Sholl‐based quantification in cortex and hippocampus: Total process length, number of branches, number of junctions (branch points), maximum branch length, and number of end‐points. Data are presented as mean ± SEM; statistical significance was assessed by unpaired Student's *t*‐test or ANOVA. ns, not significant.

MAFG knockdown (sh‐MAFG) markedly enhanced Aβ internalization relative to sh‐NC controls (Figure 6B,C; *p* = 0.0286; Figure S7A; Table S15), reduced secretion of IL‐1β, IL‐6, and TNF‐α (Figure 6D; Table S16), and restored expression of Aβ‐clearance/scavenger receptor genes while suppressing pro‐inflammatory programs (Figure 6E), consistent with the receptor repression observed in MAFG‐high progenitors (Figure 2J). MAFG therefore simultaneously constrains Aβ uptake and reinforces an inflammatory phenotype.

To establish in vivo relevance, 6‐month‐old APP/PS1 recipients received serial transfers of sh‐MAFG or sh‐NC cells (Figure 6F). Flow cytometry confirmed durable knockdown in the transferred compartment, with reduced MAFG median and mean fluorescence intensity and a lower MAFG^+^ fraction of CD45^+^ cells (Figure 6G,H; *p* = 0.0005, 0.0016 and 0.0074; Figure S7B). Recipients of sh‐MAFG cells showed improved spatial working memory in the Y‐maze (Figure 6I–K; Arm C, *p* = 0.000113; novel‐arm entries, *p* = 0.0342; Figure S7C–I). In the Morris water maze, sh‐MAFG recipients learned the platform location faster (Figure 6M; *p* < 0.0001), spent more time in the target quadrant (Figure 6N; *p* = 0.0001) and had shorter escape latencies (Figure 6O; *p* = 0.0420; heatmaps, Figure 6L; Figure S7J–M).

Neuropathology paralleled the behavioral rescue. 4G8 immunostaining revealed reduced plaque burden in sh‐MAFG recipients (Figure 6P), quantified as lower plaque load (Figure 6Q; *p* = 0.0001) and mean plaque area (*p* = 0.0212), with a concordant non‐significant trend in plaque count (*p* = 0.0608) and plaque density (*p* = 0.0639); this was corroborated by Methoxy‐X04 histochemistry (Figure 6R,S; Figure S8A; density, *p* = 0.0017; count, *p* = 0.0018; load, *p* = 0.0098). Microglial analysis showed reduced overall microgliosis together with a morphological shift toward an amoeboid phenotype: IBA1^+^ cell density was lower in cortex (*p* = 0.0421) and hippocampus (*p* < 0.0001), the IBA1^+^ area fraction was lower in hippocampus (*p* = 0.0093), and the remaining microglia showed larger soma area, higher circularity and solidity, smaller territory area and a lower morphology index in both regions (Figure 6T). Skeleton‐ and Sholl‐based reconstruction confirmed shorter total process length, fewer junctions, shorter maximum branch length and fewer end‐points (Figure 6U,V; all *p* < 0.05; average branch length in Figure S8B). Brain Aβ1–42 ELISA confirmed reduced amyloid in cortex and hippocampus (Figure S8C; *p* < 0.0001). Collectively, these data demonstrate that MAFG restrains peripheral Aβ clearance and promotes neuroinflammation, and that silencing MAFG in peripheral immune cells is sufficient to reduce central amyloid burden, remodel microglial responses and preserve cognition.

## 4 Discussion

Approximately half of septic patients develop sepsis‐associated encephalopathy during intensive care, and a substantial subset experiences cognitive dysfunction for years afterwards [47]. Sepsis accelerates cognitive decline in APP/PS1 mice with increased insoluble Aβ deposition [2], and peripheral myeloid cells and their metabolic reprogramming have been implicated in this cross‐barrier cascade [23, 48], yet the molecular mechanisms remain poorly defined.

Our atlas showed a myeloid‐skewed septic landscape—expanded monocyte/macrophage and platelet lineages with relative lymphoid reduction [49]—consistent with emergency granulopoiesis [21, 50, 51], whereas AD samples were lymphoid‐dominated with altered T and NK states enriched for AD‐risk genes [52, 53, 54, 55]. This between‐state difference [56, 57, 58] supports stage‐specific targeting.

Within this landscape we identified APP‐expressing CMPs, DCs, and platelets as an APP‐enriched compartment. Importantly, APP expression alone does not establish pathological Aβ production or a contribution to neurodegeneration; this is particularly relevant for platelets, in which APP is physiologically abundant and serves hemostatic and trophic functions. We therefore avoid framing these cells as a definitive amyloidogenic reservoir and interpret them as transcriptionally APP‐enriched populations of interest. Platelets are nonetheless a major source of circulating APP [59, 60], the bone marrow has been implicated in endogenous Aβ accumulation [61], and monocyte‐derived macrophages exert dual roles in Aβ clearance [12, 62]; under acute inflammatory stimuli this compartment may release IL‐1β, IL‐6, and chemotactic signals contributing to microglial dysfunction and synaptic impairment [6, 63].

Integrating SCENIC regulon activity across disease states—supported by an exploratory pseudotime ordering used only for hypothesis generation—identified 13 transcription factors whose activity was highest in sepsis‐derived CMPs and, separately, in AD‐derived terminal DCs and platelets. MAFG showed cross‐cohort diagnostic and prognostic value, and its early upregulation in CMPs coincided with reduced expression of canonical Aβ scavenger receptors (TLR2, CD33, MSR1), consistent with the peripheral sink hypothesis, in which the mononuclear phagocyte system facilitates efflux of brain‐derived Aβ [12]. These receptors are established mediators of myeloid Aβ handling: CD33 restrains Aβ uptake and its deletion lowers plaque burden in APP/PS1 mice [64], and MSR1/SCARA1 mediates Aβ clearance by mononuclear phagocytes [65]. We therefore moved beyond this correlation to directly test the functional consequences of MAFG activity in peripheral immune cells, as described below. Between‐state analysis further suggested distinct epigenetic programs at the progenitor level, consistent with the maladaptive arm of trained immunity [66, 67, 68, 69, 70] and with plasma proteomic overlap of neurodegenerative biomarkers between sepsis and AD [56, 71, 72].

Mechanistically, MAFG is a redox‐sensitive transcription factor that activates LCN2 to inhibit ferroptosis [73, 74] and regulates Hmox1 and Nqo1 via the FXR pathway [75]. Consistently, flow cytometry across CLP, APP/PS1, and composite models showed compartment‐specific expansion of MAFG^+^ CMPs, DCs, and platelets, paralleled by elevated MDA and transient T‐AOC compensation followed by depletion—a transition from acute oxidative response to chronic oxidative exhaustion, in line with the role of systemic oxidative stress in hippocampal neuroinflammation and synaptic dysfunction [13, 76, 77, 78]. Critically, superimposing CLP on APP/PS1 mice aggravated cerebral amyloid deposition and microglial activation and worsened recognition and spatial working memory without affecting locomotion, indicating that this peripheral remodeling is accompanied by measurable central aggravation rather than remaining a purely systemic phenomenon.

Because behavioral testing was performed 30 days after CLP—only after the acute septic illness had resolved and after the entire period of acute mortality, with body weight, food intake, sepsis score and home‐cage activity recovered to the sham range (Figure S10)—the cognitive deficits in the composite model are unlikely to be explained by transient sepsis‐induced sickness behavior. They are not, however, specific to AD. Recognition and spatial‐memory impairment of this kind is also characteristic of long‐term sepsis‐associated encephalopathy, and the present design cannot separate the two. The amyloid and microglial findings indicate that superimposed sepsis aggravates AD‐like neuropathology in APP/PS1 mice, whereas the behavioral phenotype is best interpreted as reflecting both persistent post‐sepsis impairment and exacerbation of AD‐like pathology. Distinguishing these components would require parallel testing of wild‐type CLP animals and cognitive measures with greater specificity for amyloid‐driven deficits.

To establish causation, we manipulated MAFG directly. Silencing MAFG enhanced Aβ internalization, de‐repressed scavenger receptors, and suppressed LPS‐induced cytokine production, positioning MAFG upstream of the myeloid clearance program governed by CD33 and MSR1 [64, 65]. Adoptive transfer of MAFG‐silenced cells into APP/PS1 recipients reduced amyloid burden, attenuated microglial reactivity, and preserved cognition, aligning with reports that peripherally derived monocytes counter amyloid pathogenesis [79] and that grafted peripheral myeloid cells with enhanced scavenger‐receptor expression reduce plaque burden and preserve cognition [80]. Together, these findings move MAFG beyond a correlative marker to a causal, targetable node.

Several limitations remain. Definitive dissection of the underlying transcriptional program will require knockout and rescue, cell‐type‐specific manipulation, and pharmacological targeting of the MAFG–scavenger‐receptor and MAFG–LCN2 axes. The integrated datasets derive from independent, cross‐sectional sepsis and AD individuals differing in age, inflammatory status, and treatment, and no longitudinal cohort of sepsis survivors was available. Fitting a pseudotime trajectory across two unrelated clinicopathological entities cannot recover a genuine temporal continuum and is best regarded as a computational interpolation; it was therefore used only for hypothesis generation, and every primary conclusion rests on trajectory‐independent evidence—between‐state differential expression, cross‐cohort ROC and survival analyses, and the loss‐of‐function and adoptive‐transfer experiments. To address demographic mismatch, donor age was compared across disease states and did not differ significantly (Kruskal–Wallis *p* = 0.456), and the core differential‐expression analyses were repeated with age and sex as covariates. Age adjustment left the differential‐expression structure essentially unchanged (genome‐wide log2 fold‐change correlation *r* = 0.98), the four signature genes retained their effect size and significance, MAFG expression did not track donor age within any disease group, and the signature was reproduced in an age‐matched sub‐cohort (Figure S9). SCENIC likewise infers association rather than causal regulation, and the route by which peripheral reprogramming influences the CNS, including blood–brain barrier transmigration and Aβ transport, was not directly traced. Finally, larger prospective cohorts with cognitive assessment are needed to validate the signature, and the long‐term safety of targeting the MAFG–LCN2 axis warrants investigation.

## 5 Conclusion

Peripheral immune states differ between sepsis and AD and are captured by a MAFG‐centered four‐gene signature with cross‐cohort diagnostic and prognostic value. Loss‐of‐function and adoptive‐transfer experiments establish MAFG as a causal, redox‐sensitive regulatory node that constrains peripheral Aβ clearance and promotes neuroinflammation, identifying it as a candidate biomarker and therapeutic target for cognitive‐risk stratification and intervention in sepsis survivors.

## Author Contributions

W.Z. and X.X. designed the study. X.X. performed data curation and formal analysis, and drafted the manuscript. W.Z. reviewed and edited the manuscript. T.L., J.Y., Z.G., Lt.W., and Lj.W. contributed to methodology and data acquisition. W.Z. and Z.F. acquired funding. All authors approved the final version of the manuscript for publication.

## Funding

This work was supported by the National Natural Science Foundation of China (82173262 to W.Z.), the Sichuan Science and Technology Program (2024NSFSC1536 to Z.F.).

## Ethics Statement

The observational study using human blood samples for measurement of adaptive immunity‐related gene expression was approved by Sichuan University on March 5, 2021 (Approval No. MR 2021_S053). Informed consent was obtained from all subjects. All animal treatments were authorized by the Laboratory Animal Ethical Committee of West China Hospital, Sichuan University (Approval No. 2021‐S‐012). All animal handling and procedures complied with the Guidelines for Ethical Review of Laboratory Animal Welfare in China and the Guide for the Care and Use of Laboratory Animals by the National Research Council.

## Conflicts of Interest

The authors declare no conflicts of interest.

## Supporting information


**Figure S1:** Lineage‐level marker characterization, disease‐specific composition, and topographical distribution of peripheral immune cells.
**Figure S2:** Detailed annotation, marker characterization, and compositional analysis of the 14 functional subtypes derived from APP‐enriched meta‐clusters.
**Figure S3:** Hierarchical sub‐clustering identifies CMPs, DCs, and platelets as the principal peripheral APP‐expressing populations.
**Figure S4:** Exploratory pseudotemporal ordering suggests differences in epigenetic and AD‐risk gene programs between cell states. The ordering was derived from independent, cross‐sectional sepsis and AD cohorts and is presented for hypothesis generation only; it does not represent progression over time.
**Figure S5:** Uncropped Western blots, flow cytometry gating strategy, and independent replication of the recognition‐memory phenotype.
**Figure S6:** Representative amyloid/microglia co‐staining and supplementary morphometric quantification in APP/PS1 and APP/PS1 + CLP mice.
**Figure S7:** Flow cytometric verification and supplementary behavioral parameters in the adoptive‐transfer cohort.
**Figure S8:** Supplementary amyloid pathology in the adoptive‐transfer cohort.
**Figure S9:** Age and sex adjustment does not alter the MAFG‐centered signature.
**Figure S10:** Physiological recovery precedes behavioral testing in the composite APP/PS1 + CLP model.

**Data S1:** Supplementary Methods.

**Table S1:** Information on the GEO datasets used in the study for sepsis and Alzheimer's disease.
**Table S2:** Antibodies, reagents, and resources used in this study.
**Table S3:** Per‐sample quality‐control metrics and cell numbers for the integrated single‐cell RNA‐sequencing datasets.
**Table S4:** Marker genes for the 28 clusters and 9 major immune lineages.
**Table S5:** Cluster‐level statistical comparison of immune‐cell abundance across the healthy control, Alzheimer's disease, and sepsis cohorts.
**Table S6:** Top DEGs for the 14 functional subtypes.
**Table S7:** GO/KEGG enrichment of modules and 13 core TFs.
**Table S8:** AD‐risk gene list and 29 ad blood biomarkers.
**Table S9:** Pseudo‐bulk DEGs across disease states (CMP/DC/platelet).
**Table S10:** SCENIC regulons and the 13 core transcription factors.
**Table S11:** Clinical characteristics and peripheral‐blood expression of candidate genes in the in‐house sepsis cohort (GCS‐stratified).
**Table S12:** Baseline clinical characteristics of the in‐house cohorts.
**Table S13:** Diagnostic AUC (peripheral, central, and 4‐gene signature).
**Table S14:** CERAD/Braak neuropathological scoring and expression of candidate genes in the brain‐tissue cohort (GSE135838).
**Table S15:** Source data: RT‐qPCR and Aβ‐uptake assay.
**Table S16:** Source data: oxidative stress, cytokine & Aβ1‐42 ELISA.
**Table S17:** Source data: flow cytometry MAFG^+^ subpopulations.
**Table S18:** Source data: behavioral tests (NOR, Y‐maze, MWM).
**Table S19:** Source data: plaque & Iba1/microglial morphometry.

## Data Availability

The data that support the findings of this study are available in the Genome Sequence Archive (GSA) for Humans at https://ngdc.cncb.ac.cn/gsa‐human/browse/HRA002988, reference number HRA002988. These data were derived from the following resources available in the public domain: Gene Expression Omnibus (GEO), https://www.ncbi.nlm.nih.gov/geo/, accession numbers GSE151263, GSE167363, GSE135838, GSE65682, GSE226602, and GSE181279.

## References

[1] X. Zhang , K. Huang , Z. Wu , et al., “Sepsis‐Associated Encephalopathy: Unraveling Molecular Mechanisms, Emerging Therapeutics, and Translational Frontiers,” Pharmacology & Therapeutics 278 (2026): 108971. 10.1016/j.pharmthera.2025.108971 41456741

[2] V. V. Giridharan , C. S. G. Catumbela , C. H. R. Catalao , et al., “Sepsis Exacerbates Alzheimer's Disease Pathophysiology, Modulates the Gut Microbiome, Increases Neuroinflammation and Amyloid Burden,” Molecular Psychiatry 28 (2023): 4463–4473. 10.1038/s41380-023-02172-2 PMC10926876 37452088

[3] L. He , X. Duan , S. Li , R. Zhang , X. Dai , and M. Lu , “Unveiling the Role of Astrocytes in Postoperative Cognitive Dysfunction,” Ageing Research Reviews 95 (2024): 102223. 10.1016/j.arr.2024.102223 38325753

[4] N. K. Navalpur Shanmugam , F. Zamudio , D. K. Vijaya Kumar , et al., “Acute Experimental Colitis in 5xFAD Alzheimer's Disease Mice Leads to Enhanced Monocyte Infiltration Into the Brain Accompanied by Reduced Beta‐Amyloid Deposition,” Alzheimer's & Dementia 21 (2025): e70292. 10.1002/alz.70292 PMC12130571 40457749

[5] M. Calvo‐Rodriguez , E. K. Kharitonova , A. C. Snyder , et al., “Real‐Time Imaging of Mitochondrial Redox Reveals Increased Mitochondrial Oxidative Stress Associated With Amyloid Beta Aggregates In Vivo in a Mouse Model of Alzheimer's Disease,” Molecular Neurodegeneration 19 (2024): 6. 10.1186/s13024-024-00702-2 PMC10797952 38238819

[6] H. Y. Chung , J. Wickel , N. Hahn , et al., “Microglia Mediate Neurocognitive Deficits by Eliminating C1q‐Tagged Synapses in Sepsis‐Associated Encephalopathy,” Science Advances 9 (2023): eabq7806. 10.1126/sciadv.abq7806 PMC10219600 37235660

[7] C. Ge , W. Chen , L. Zhang , Y. Ai , Y. Zou , and Q. Peng , “Chemogenetic Activation of the HPC‐mPFC Pathway Improves Cognitive Dysfunction in Lipopolysaccharide‐Induced Brain Injury,” Theranostics 13 (2023): 2946–2961. 10.7150/thno.82889 PMC10240833 37284451

[8] B. Grunewald , J. Wickel , N. Hahn , et al., “Targeted Rescue of Synaptic Plasticity Improves Cognitive Decline in Sepsis‐Associated Encephalopathy,” Molecular Therapy 32 (2024): 2113–2129. 10.1016/j.ymthe.2024.05.001 PMC11286813 38788710

[9] Y. J. Kang , S. J. Hyeon , A. McQuade , et al., “Neurotoxic Microglial Activation via IFNgamma‐Induced Nrf2 Reduction Exacerbating Alzheimer's Disease,” Advanced Science (Weinheim) 11 (2024): e2304357. 10.1002/advs.202304357 PMC11132036 38482922

[10] N. Yu , M. Pasha , and J. J. E. Chua , “Redox Changes and Cellular Senescence in Alzheimer's Disease,” Redox Biology 70 (2024): 103048. 10.1016/j.redox.2024.103048 PMC10840360 38277964

[11] Y. Zhang , J. Xin , D. Zhao , et al., “Magnesium Hexacyanoferrate Mitigates Sepsis‐Associated Encephalopathy Through Inhibiting Microglial Activation and Neuronal Cuproptosis,” Biomaterials 321 (2025): 123279. 10.1016/j.biomaterials.2025.123279 40164040

[12] X. Huang , C. Fowler , Y. Li , et al., “Clearance and Transport of Amyloid β by Peripheral Monocytes Correlate With Alzheimer's Disease Progression,” Nature Communications 15 (2024): 7998. 10.1038/s41467-024-52396-1 PMC11393069 39266542

[13] X. W. Mo , D. K. Min , and J. Kim , “Recent Advances in ROS‐Modulating Materials for Sepsis Treatment,” Journal of Controlled Release 393 (2026): 114779. 10.1016/j.jconrel.2026.114779 41780687

[14] E. H. A. Michels , J. M. Butler , T. D. Y. Reijnders , et al., “Association Between Age and the Host Response in Critically Ill Patients With Sepsis,” Critical Care 26 (2022): 385. 10.1186/s13054-022-04266-9 PMC9747080 36514130

[15] H. R. Li , Q. Liu , C. L. Zhu , et al., “Beta‐Nicotinamide Mononucleotide Activates NAD+/SIRT1 Pathway and Attenuates Inflammatory and Oxidative Responses in the Hippocampus Regions of Septic Mice,” Redox Biology 63 (2023): 102745. 10.1016/j.redox.2023.102745 PMC10206198 37201414

[16] Q. Cheng , J. Nolz , T. Karr , et al., “Gut Proteome and Microbiome Alterations: Analysis of Transverse Colon Samples From Pathologically Confirmed Alzheimer's Disease Patients,” Alzheimers Dement 22 (2026): e71021. 10.1002/alz.71021 PMC12796839 41527485

[17] Y. Zhao , W. Zhao , X. Chai , et al., “Reshaping the Gut Microbiota: A Novel Oppinion of Eucommiae Cortex Polysaccharide Alleviate Learning and Memory Impairments in Alzheimer's Disease,” Journal of Advanced Research 80 (2026): 219–237. 10.1016/j.jare.2025.04.025 PMC12869218 40252828

[18] L. Zhao , Y. Huang , Z. Ye , et al., “Short‐Chain Fatty Acids Attenuate Sepsis‐Induced Gut Dysbiosis and Hippocampal Neuroinflammation via NLRP6 Inflammasome Activation in Mice,” International Journal of Surgery 112 (2026): 570–582. 10.1097/JS9.0000000000003554 PMC12825554 41159411

[19] Y. Wu , Y. Zhang , Y. Yu , et al., “Small Intestinal Gammadelta T17 Cells Promote SAE Through STING/C1q‐Induced Microglial Synaptic Pruning in Male Mice,” Nature Communications 16 (2025): 6779. 10.1038/s41467-025-62181-3 PMC12287374 40702081

[20] R. Q. Yao , Z. X. Li , L. X. Wang , et al., “Single‐Cell Transcriptome Profiling of the Immune Space‐Time Landscape Reveals Dendritic Cell Regulatory Program in Polymicrobial Sepsis,” Theranostics 12 (2022): 4606–4628. 10.7150/thno.72760 PMC9254255 35832091

[21] A. J. Kwok , A. Allcock , R. C. Ferreira , et al., “Neutrophils and Emergency Granulopoiesis Drive Immune Suppression and an Extreme Response Endotype During Sepsis,” Nature Immunology 24 (2023): 767–779. 10.1038/s41590-023-01490-5 37095375

[22] G. Tang , W. Xing , L. Zhu , et al., “Illustrating the Functional Heterogeneity of M‐MDSCs to Predict Sepsis Outcomes,” Journal of Advanced Research 88 (2026): 807–824.10.1016/j.jare.2026.01.00841490841

[23] N. Hua , L. Kong , L. Fan , et al., “Identification of CD38(High) Monocyte as a Candidate Diagnostic Biomarker and Therapeutic Target for Sepsis,” Advanced Science 12 (2025): e2500457. 10.1002/advs.202500457 PMC12199442 40145840

[24] X. F. Sun , W. C. Luo , S. Q. Huang , et al., “Immune‐Cell Signatures of Persistent Inflammation, Immunosuppression, and Catabolism Syndrome After Sepsis,” Medicus 6 (2025): 100569. 10.1016/j.medj.2024.12.003 39824181

[25] R. Q. Yao , P. Y. Zhao , Z. X. Li , et al., “Single‐Cell Transcriptome Profiling of Sepsis Identifies HLA‐DR(Low)S100A(High) Monocytes With Immunosuppressive Function,” Military Medical Research 10 (2023): 27. 10.1186/s40779-023-00462-y PMC10278311 37337301

[26] A. C. Bustamante , K. Opron , W. J. Ehlenbach , et al., “Transcriptomic Profiles of Sepsis in the Human Brain,” American Journal of Respiratory and Critical Care Medicine 201 (2020): 861–863. 10.1164/rccm.201909-1713LE PMC7124721 31940219

[27] R. B. E. van Amstel , J. N. Kennedy , B. P. Scicluna , et al., “Uncovering Heterogeneity in Sepsis: A Comparative Analysis of Subphenotypes,” Intensive Care Medicine 49 (2023): 1360–1369. 10.1007/s00134-023-07239-w PMC10622359 37851064

[28] J. T. Leek , W. E. Johnson , H. S. Parker , A. E. Jaffe , and J. D. Storey , “The Sva Package for Removing Batch Effects and Other Unwanted Variation in High‐Throughput Experiments,” Bioinformatics 28 (2012): 882–883. 10.1093/bioinformatics/bts034 PMC3307112 22257669

[29] H. Xu and J. Jia , “Single‐Cell RNA Sequencing of Peripheral Blood Reveals Immune Cell Signatures in Alzheimer's Disease,” Frontiers in Immunology 12 (2021): 645666. 10.3389/fimmu.2021.645666 PMC8382575 34447367

[30] A. Ramakrishnan , N. Piehl , B. Simonton , et al., “Epigenetic Dysregulation in Alzheimer's Disease Peripheral Immunity,” Neuron 112 (2024): 1235–1248.e5. 10.1016/j.neuron.2024.01.013 PMC11031321 38340719

[31] X. Qiu , J. Li , J. Bonenfant , et al., “Dynamic Changes in Human Single‐Cell Transcriptional Signatures During Fatal Sepsis,” Journal of Leukocyte Biology 110 (2021): 1253–1268. 10.1002/JLB.5MA0721-825R PMC8629881 34558746

[32] Y. Jiang , B. R. Rosborough , J. Chen , et al., “Single Cell RNA Sequencing Identifies an Early Monocyte Gene Signature in Acute Respiratory Distress Syndrome,” JCI Insight 5 (2020): e135678. 10.1172/jci.insight.135678 PMC7406263 32554932

[33] Y. Hao , T. Stuart , M. H. Kowalski , et al., “Dictionary Learning for Integrative, Multimodal and Scalable Single‐Cell Analysis,” Nature Biotechnology 42 (2024): 293–304. 10.1038/s41587-023-01767-y PMC10928517 37231261

[34] L. Zhang , X. Yu , L. Zheng , et al., “Lineage Tracking Reveals Dynamic Relationships of T Cells in Colorectal Cancer,” Nature 564 (2018): 268–272. 10.1038/s41586-018-0694-x 30479382

[35] C. Trapnell , D. Cacchiarelli , J. Grimsby , et al., “The Dynamics and Regulators of Cell Fate Decisions Are Revealed by Pseudotemporal Ordering of Single Cells,” Nature Biotechnology 32 (2014): 381–386. 10.1038/nbt.2859 PMC4122333 24658644

[36] S. Aibar , C. B. González‐Blas , T. Moerman , et al., “SCENIC: Single‐Cell Regulatory Network Inference and Clustering,” Nature Methods 14 (2017): 1083–1086. 10.1038/nmeth.4463 PMC5937676 28991892

[37] A. Subramanian , P. Tamayo , V. K. Mootha , et al., “Gene Set Enrichment Analysis: A Knowledge‐Based Approach for Interpreting Genome‐Wide Expression Profiles,” Proceedings of the National Academy of Sciences of the United States of America 102 (2005): 15545–15550. 10.1073/pnas.0506580102 PMC1239896 16199517

[38] X. Robin , N. Turck , A. Hainard , et al., “pROC: An Open‐Source Package for R and S+ to Analyze and Compare ROC Curves,” BMC Bioinformatics 12 (2011): 77. 10.1186/1471-2105-12-77 PMC3068975 21414208

[39] H. Yao , Y. Zhou , T. Li , et al., “Bioinformatic Identification and Experiment Validation Revealed That ACTG1 Is a Promising Prognostic Signature and Therapeutic Target for Sepsis,” Journal of Leukocyte Biology 114 (2023): 325–334. 10.1093/jleuko/qiad072 37368839

[40] D. Rittirsch , M. S. Huber‐Lang , M. A. Flierl , and P. A. Ward , “Immunodesign of Experimental Sepsis by Cecal Ligation and Puncture,” Nature Protocols 4 (2009): 31–36. 10.1038/nprot.2008.214 PMC2754226 19131954

[41] A. C. Yang , R. T. Vest , F. Kern , et al., “A Human Brain Vascular Atlas Reveals Diverse Mediators of Alzheimer's Risk,” Nature 603 (2022): 885–892. 10.1038/s41586-021-04369-3 PMC9635042 35165441

[42] P. Y. Sun , J. Liu , J. N. Hu , et al., “Rejuvenation of Peripheral Immune Cells Attenuates Alzheimer's Disease‐Like Pathologies and Behavioral Deficits in a Mouse Model,” Science Advances 10 (2024): eadl1123. 10.1126/sciadv.adl1123 PMC11135428 38809977

[43] A. B. Niculescu , H. Le‐Niculescu , K. Roseberry , et al., “Blood Biomarkers for Memory: Toward Early Detection of Risk for Alzheimer Disease, Pharmacogenomics, and Repurposed Drugs,” Molecular Psychiatry 25 (2020): 1651–1672. 10.1038/s41380-019-0602-2 PMC7387316 31792364

[44] M. A. Wheeler , I. C. Clark , E. C. Tjon , et al., “MAFG‐Driven Astrocytes Promote CNS Inflammation,” Nature 578 (2020): 593–599. 10.1038/s41586-020-1999-0 PMC8049843 32051591

[45] H. G. Lee , J. M. Rone , Z. Li , et al., “Disease‐Associated Astrocyte Epigenetic Memory Promotes CNS Pathology,” Nature 627 (2024): 865–872. 10.1038/s41586-024-07187-5 PMC11016191 38509377

[46] J. E. Burda , T. M. O'Shea , Y. Ao , et al., “Divergent Transcriptional Regulation of Astrocyte Reactivity Across Disorders,” Nature 606 (2022): 557–564. 10.1038/s41586-022-04739-5 PMC10027402 35614216

[47] T. Manabe and M. T. Heneka , “Cerebral Dysfunctions Caused by Sepsis During Ageing,” Nature Reviews. Immunology 22 (2022): 444–458. 10.1038/s41577-021-00643-7 PMC8582341 34764472

[48] X. Wang , J. Ning , L. Zhou , et al., “Targeting Matrix Metalloproteinase‐9 to Alleviate T Cell Exhaustion and Improve Sepsis Prognosis,” Research 8 (2025): 0996. 10.34133/research.0996 PMC12645450 41306770

[49] D. M. Bonal , A. Oakes , A. Chorzalska , et al., “HiJAKing the Hematopoietic System: A Low‐Frequency JAK2V617F Clone Drives Myeloproliferative Neoplasm Pathology,” Blood 146 (2025): 2428–2442. 10.1182/blood.2024027125 PMC13077795 40729727

[50] E. M. Pietras and J. DeGregori , “Dangerous Liaisons Between Tet2 Mutation, Inflammatory Monocytes, and Leukemogenesis,” Cancer Discovery 12 (2022): 2234–2236. 10.1158/2159-8290.CD-22-0846 36196576

[51] X. Wu , S. Liu , W. Hu , et al., “From Defense to Dysfunction: Decoding the Paradox of Emergency Granulopoiesis in Sepsis Pathogenesis,” Research (Wash D C) 8 (2025): 1011. 10.34133/research.1011 PMC12722637 41446870

[52] L. van Olst , A. Kamermans , S. Halters , et al., “Adaptive Immune Changes Associate With Clinical Progression of Alzheimer's Disease,” Molecular Neurodegeneration 19 (2024): 38. 10.1186/s13024-024-00726-8 PMC11044380 38658964

[53] E. Terrabuio and G. Constantin , “APOE4 Affects Neutrophil‐Microglia Crosstalk in Alzheimer's Disease,” Trends in Immunology 45 (2024): 726–728. 10.1016/j.it.2024.09.002 39322476

[54] C. Fernandez Zapata , G. Giacomello , E. J. Spruth , et al., “Differential Compartmentalization of Myeloid Cell Phenotypes and Responses Towards the CNS in Alzheimer's Disease,” Nature Communications 13 (2022): 7210. 10.1038/s41467-022-34719-2 PMC9684147 36418303

[55] O. Butovsky , N. Rosenzweig , K. L. Kleemann , et al., “Immune Dysfunction in Alzheimer Disease,” Nature Reviews: Neuroscience 27 (2026): 196–218. 10.1038/s41583-025-00997-0 PMC13010386 41315874

[56] K. A. Walker , L. M. Le Page , N. Terrando , et al., “The Role of Peripheral Inflammatory Insults in Alzheimer's Disease: A Review and Research Roadmap,” Molecular Neurodegeneration 18 (2023): 37. 10.1186/s13024-023-00627-2 PMC10240487 37277738

[57] M. S. Paladini , B. A. Yang , K. A. Torkenczy , et al., “Fate Mapping of Peripherally‐Derived Macrophages After Traumatic Brain Injury in Mice Reveals a Long‐Lasting Population With a Distinct Transcriptomic Signature,” Nature Communications 16 (2025): 8898. 10.1038/s41467-025-63952-8 PMC12504527 41057304

[58] M. Shankar‐Hari , T. Calandra , M. P. Soares , et al., “Reframing Sepsis Immunobiology for Translation: Towards Informative Subtyping and Targeted Immunomodulatory Therapies,” Lancet Respiratory Medicine 12 (2024): 323–336. 10.1016/S2213-2600(23)00468-X PMC11025021 38408467

[59] M. G. Carbone , G. Pagni , C. Tagliarini , et al., “Can Platelet Activation Result in Increased Plasma Abeta Levels and Contribute to the Pathogenesis of Alzheimer's Disease?,” Ageing Research Reviews 71 (2021): 101420. 10.1016/j.arr.2021.101420 34371202

[60] G. Rodriguez Moore , I. Melo‐Escobar , D. Stegner , and O. Bracko , “One Immune Cell to Bind Them All: Platelet Contribution to Neurodegenerative Disease,” Molecular Neurodegeneration 19 (2024): 65. 10.1186/s13024-024-00754-4 PMC11438031 39334369

[61] J. Zhang , W. Fang , H. Liu , et al., “Bone Marrow B Lymphopoiesis Accelerates Early Cerebral Amyloid Pathology,” Signal Transduction and Targeted Therapy 10 (2025): 312. 10.1038/s41392-025-02419-0 PMC12443962 40962797

[62] H. Ben‐Yehuda , M. Arad , J. M. Peralta Ramos , et al., “Key Role of the CCR2‐CCL2 Axis in Disease Modification in a Mouse Model of Tauopathy,” Molecular Neurodegeneration 16 (2021): 39. 10.1186/s13024-021-00458-z PMC8234631 34172073

[63] A. B. Lopez‐Rodriguez , E. Hennessy , C. L. Murray , et al., “Acute Systemic Inflammation Exacerbates Neuroinflammation in Alzheimer's Disease: IL‐1beta Drives Amplified Responses in Primed Astrocytes and Neuronal Network Dysfunction,” Alzheimer's & Dementia 17 (2021): 1735–1755. 10.1002/alz.12341 PMC8874214 34080771

[64] A. Griciuc , A. Serrano‐Pozo , A. R. Parrado , et al., “Alzheimer's Disease Risk Gene CD33 Inhibits Microglial Uptake of Amyloid Beta,” Neuron 78 (2013): 631–643. 10.1016/j.neuron.2013.04.014 PMC3706457 23623698

[65] D. Frenkel , K. Wilkinson , L. Zhao , et al., “Scara1 Deficiency Impairs Clearance of Soluble Amyloid‐Beta by Mononuclear Phagocytes and Accelerates Alzheimer's‐Like Disease Progression,” Nature Communications 4 (2013): 2030. 10.1038/ncomms3030 PMC3702268 23799536

[66] A. Ziogas , B. Novakovic , L. Ventriglia , et al., “Long‐Term Histone Lactylation Connects Metabolic and Epigenetic Rewiring in Innate Immune Memory,” Cell 188 (2025): 2992–3012.e16. 10.1016/j.cell.2025.03.048 40318634

[67] A. Ziogas , M. Bruno , R. van der Meel , W. J. M. Mulder , and M. G. Netea , “Trained Immunity: Target for Prophylaxis and Therapy,” Cell Host & Microbe 31 (2023): 1776–1791. 10.1016/j.chom.2023.10.015 37944491

[68] Y. Pan , J. Li , X. Xia , et al., “Beta‐Glucan‐Coupled Superparamagnetic Iron Oxide Nanoparticles Induce Trained Immunity to Protect Mice Against Sepsis,” Theranostics 12 (2022): 675–688. 10.7150/thno.64874 PMC8692910 34976207

[69] J. Jia , W. Ji , N. Xiong , J. Lin , and Q. Yang , “Trained Immunity Using Probiotics and Inactivated Pathogens Enhances Resistance to *Salmonella enterica* Serovar Typhimurium Infection by Activating the cGAS‐STING Signal Pathway in Mice and Chickens,” Journal of Advanced Research 79 (2026): 491–504. 10.1016/j.jare.2025.03.011 PMC12766225 40086629

[70] J. Weng , Y. Yang , C. Li , et al., “Locus Coeruleus‐Hippocampus Noradrenergic Activation Alleviates Sepsis‐Associated Encephalopathy by Promoting Astrocytic AQP4‐Related Autophagy via Alpha(2)A‐AR,” Journal of Advanced Research (2026). 10.1016/j.jare.2026.03.024 41831677

[71] L. Xie , S. R. Das , Y. Li , et al., “A Multi‐Cohort Study of Longitudinal and Cross‐Sectional Alzheimer's Disease Biomarkers in Cognitively Unimpaired Older Adults,” Alzheimer's & Dementia 21 (2025): e14492. 10.1002/alz.14492 PMC11848397 39868491

[72] E. Fertan , G. Meisl , and D. Klenerman , “Small‐Diffusible Aggregates, Plaques, Tangles, and Dynamic Equilibria: Untangling Alzheimer's Disease,” Alzheimer's & Dementia 21 (2025): e70462. 10.1002/alz.70462 PMC12226248 40611371

[73] Y. Deng , L. Lu , D. Zhu , et al., “MafG/MYH9‐LCN2 Axis Promotes Liver Fibrosis Through Inhibiting Ferroptosis of Hepatic Stellate Cells,” Cell Death and Differentiation 31 (2024): 1127–1139. 10.1038/s41418-024-01322-5 PMC11369194 38871948

[74] X. Zhang , X. Hu , C. Fu , et al., “Novel MAFG‐METTL14‐SCD1 Axis Regulates Lipid Metabolism Mediating Choroidal Melanoma Distant Metastasis,” Journal of Experimental & Clinical Cancer Research 44 (2025): 334. 10.1186/s13046-025-03595-1 PMC12751620 41316357

[75] D. H. Kim , H. I. Choi , J. S. Park , et al., “Farnesoid X Receptor Protects Against Cisplatin‐Induced Acute Kidney Injury by Regulating the Transcription of Ferroptosis‐Related Genes,” Redox Biology 54 (2022): 102382. 10.1016/j.redox.2022.102382 PMC9241134 35767918

[76] H. Qu , J. Wu , Y. Pan , et al., “Biomimetic Nanomodulator Regulates Oxidative and Inflammatory Stresses to Treat Sepsis‐Associated Encephalopathy,” ACS Nano 18 (2024): 28228–28245. 10.1021/acsnano.4c08157 39367850

[77] J. Wang , Z. Zhong , H. Luo , et al., “Modulation of Brain Immune Microenvironment and Cellular Dynamics in Systemic Inflammation,” Theranostics 15 (2025): 5153–5171. 10.7150/thno.107061 PMC12036864 40303348

[78] W. Wang , Y. Hai , X. Lu , et al., “Nanotherapeutic Macrophage‐Neuro Reprogramming Through Immunometabolic Crosstalk Mitigates Sepsis‐Induced Lung Injury and Neurologic Damage,” Advanced Science (Weinh) 13 (2026): e20665. 10.1002/advs.202520665 PMC13116209 41698047

[79] P. Yan , K. W. Kim , Q. Xiao , et al., “Peripheral Monocyte‐Derived Cells Counter Amyloid Plaque Pathogenesis in a Mouse Model of Alzheimer's Disease,” Journal of Clinical Investigation 132 (2022): e152565. 10.1172/JCI152565 PMC9151689 35511433

[80] Y. Koronyo , B. C. Salumbides , J. Sheyn , et al., “Therapeutic Effects of Glatiramer Acetate and Grafted CD115+ Monocytes in a Mouse Model of Alzheimer's Disease,” Brain 138 (2015): 2399–2422. 10.1093/brain/awv150 PMC4840949 26049087

