# Supplementary material for: MAFG as a Redox‐Sensitive Node Linking Peripheral Immune Reprogramming to Cognitive Risk in the Sepsis‐to‐Alzheimer's Disease Continuum: Single‐Cell Dissection and Adoptive‐Transfer Validation
Source: CNS Neurosci Ther. 2026 Oct 13;32(10):e71201. doi: 10.1002/cns.71201 (PMC13659262; doi:10.1002/cns.71201)
Supplement: Supplementary file 1 [file CNS-32-e71201-s001.docx]

**FIGURE | S1** Lineage-level marker characterization, disease-specific composition, and topographical distribution of peripheral immune cells. (**A**) Violin plots showing the expression of canonical marker genes used to annotate the 9 major immune lineages. (**B**) UMAP feature plots of representative lineage-defining markers (CD19, CD79A for B cells; CD68, CD14, LYZ for Mon/Mac; NKG7 for NK cells; CD2, CD3D for T/NKT cells; PPBP for platelets). (**C**) Stacked bar charts showing absolute cell counts (top two panels) and proportional abundances (bottom two panels) of the 28 clusters and 9 major lineages across individual samples in HC (n = 26), AD (n = 31), and Sepsis (n = 14) cohorts. (**D–E**) Cluster-level disease preference heatmaps showing OR (**D**) and Ro/e (**E**) values across the three disease states; symbols in E indicate enrichment preference (+, positive; +/−, neutral; −, negative). (**F**) Split UMAP projections showing the topographical clustering patterns of the 28 clusters in HC, AD, and Sepsis cohorts; arrows annotate differences in lineage abundance relative to HC. (**G**) Cluster-level statistical comparison of cell-type abundance across the three cohorts. Each dot represents an individual cluster; significance levels are indicated above (NS, not significant; *p < 0.05, **p < 0.01, ***p < 0.001). (**H**) Violin plot showing APP expression across all 28 single-cell clusters, stratified by disease state.

**FIGURE | S2** Detailed annotation, marker characterization, and compositional analysis of the 14 functional subtypes derived from APP-enriched meta-clusters. (**A**) Violin plots showing the expression of canonical markers across the 14 subtypes; dashed boxes highlight cluster-defining markers. (**B**) UMAP embedding of the 14 functional subtypes annotated with cell-type identities. (**C**) UMAP feature plots of canonical lineage markers (FCER1A, CSF3R, FCN1, CD14, VCAN, FCGR3A, LYZ, CD163 for myeloid lineages; CD68, MARCO, CLEC9A, CD3D, C1QB, KLRD1, CD79A, PPBP for additional lineage identification). (**D**) Stacked-area plot showing the proportional composition of the 14 subtypes across HC, AD, and Sepsis cohorts. (**E**) Box plots comparing the frequency of each subtype across the three cohorts (NS, not significant; *p < 0.05, **p < 0.01, ***p < 0.001, ****p < 0.0001). (**F**) Heatmap showing the top differentially expressed genes (DEGs) defining each of the 14 subtypes.

**FIGURE | S3** Hierarchical sub-clustering identifies CMPs, DCs, and platelets as the principal peripheral APP-expressing populations. (**A**) Left, split UMAP projections showing the topographies of 14 functional subtypes derived from the APP-enriched meta-clusters in HC, AD, and Sepsis cohorts; color scale indicates APP expression. Right, box plot of overall APP mRNA expression across the three cohorts (****p < 0.0001). (**B**) Violin plots showing APP expression across the 14 subtypes. The dashed-box inset highlights the AD-specific enrichment of APP in Cluster 9 (****p < 0.0001). NS, not significant; *p < 0.05, **p < 0.01, ***p < 0.001, ****p < 0.0001. (**C**) Violin plots showing the expression of canonical lineage markers across the 14 subtypes; dashed boxes mark the most discriminating markers per cluster. (**D**) UMAP embedding of the 14 functional subtypes annotated with cell-type identities (Neu, neutrophil; Macro, macrophage; Mono, monocyte; Mega, megakaryocyte; DC, dendritic cell; B, B cell; T, T cell; NK, natural killer cell). (**E**) Heatmap showing the top 10 differentially expressed genes (DEGs) defining each terminal subtype. (**F**) UMAP feature plots of representative canonical markers for platelets (PPBP, PF4, GP9, TUBB1), effector memory CD8⁺ αβ T cells (CD300A, GZMB, GNLY, PRF1), CMPs (MPO, RNASE2, FCGR1A, CD34), and CD1C⁻CD141⁻ DCs (PPP1R14B, CAMK1, PRDX4, TSPAN32).

**FIGURE | S4** Exploratory pseudotemporal ordering suggests differences in epigenetic and AD-risk gene programs between cell states. The ordering was derived from independent, cross-sectional sepsis and AD cohorts and is presented for hypothesis generation only; it does not represent progression over time. (**A–B**) Monocle-inferred pseudotime trajectories of the APP⁺ cellular compartments, annotated by terminal subtypes (**A**) and disease cohorts (**B**), with pie charts showing the proportional composition of major branches. (**C**) Cell state distribution along the trajectory, with pie charts showing state preferences in HC, Sepsis, and AD. (**D**) Transcriptomic modules along the trajectory. Left: Heatmap showing gene expression ordered by pseudotime in four modules (C1-C4). Right: GO (Biological Process) and KEGG enrichment for each module. (**E**) Heatmap showing differences in AD-risk gene expression between sepsis-derived CMPs and AD-derived DCs and platelets. (**F**) Heatmap showing the expression of epigenetic regulators (DNA/histone methylation and acetylation, chromatin organization) across the four cellular subtypes, indicating higher expression in AD-derived DCs and platelets. *p < 0.05, **p < 0.01, ***p < 0.001, ****p < 0.0001.

**FIGURE | S5** Uncropped Western blots, flow cytometry gating strategy, and independent replication of the recognition-memory phenotype. (**A**) Full-length, uncropped Western blots for Mafg and β-actin in the peripheral blood of Sham, CLP (8 h and 24 h), and APP/PS1 mice, corresponding to the cropped images in Figure 4E. (**B**) Representative gating strategy for flow cytometric identification of MAFG⁺ subsets. Bone marrow CMPs (BM-CMP) and peripheral blood CMPs (BD-CMP) were gated as live CD45⁺CD38⁻CD34⁺CD123⁺ cells; peripheral platelets (BD-Platelet) as CD41⁺ cells; and peripheral DCs (BD-DC) as CD11c⁺MHC II⁺CD14⁻ cells, with MAFG expression assessed in each population. (**C**) Experimental timeline. APP/PS1 mice underwent CLP or sham surgery on day 0 after a baseline monitoring period; body weight, food intake, murine sepsis score and home-cage activity were recorded throughout the acute and recovery phases (Figure S10). Behavioral testing (Y-maze and novel object recognition) began on day 30, after physiological recovery was confirmed, and was followed by transcardial perfusion for neuropathological analysis. (**D–H**) Independent validation of the novel object recognition (NOR) test in a separate cohort of APP/PS1 and APP/PS1 + CLP mice. Distance moved (**D**; p = 0.6901) and velocity (**E**; p = 0.0541) did not differ between groups, whereas the discrimination index (**F**; p = 0.0002) and the recognition index (**G**; p = 0.0005) were significantly reduced in APP/PS1 + CLP mice. (**H**) Representative object-exploration heatmaps. Data are presented as mean ± SEM; statistical significance was assessed by unpaired Student’s t-test. NS, not significant.

**FIGURE | S6** Representative amyloid/microglia co-staining and supplementary morphometric quantification in APP/PS1 and APP/PS1 + CLP mice. (**A**) Representative immunofluorescence images of amyloid plaques (Methoxy-X04, blue), Iba1⁺ microglia (yellow), and merged channels in APP/PS1 and APP/PS1 + CLP brains; scale bars, 50 µm. (**B–D**) Quantification of plaque number (**B**), plaque burden (**C**) and mean plaque area (**D**). **(E–G**) Microglial morphometry: number of branches per microglial cell (**E**), mean process length (**F**) and total number of junctions (**G**). Group comparisons for these measures were not statistically significant (NS), complementing the significant differences in plaque density and Iba1 burden reported in Figure 5. Data are presented as mean ± SEM; statistical significance was assessed by unpaired Student’s t-test. NS, not significant.

**FIGURE | S7** Flow cytometric verification and supplementary behavioral parameters in the adoptive-transfer cohort. (**A**) Complete flow cytometry plots of Aβ uptake (Aβ⁺) in Negative Ctrl, sh-NC and sh-MAFG cells, corresponding to the representative plots in Figure 6B. (**B**) Complete flow cytometry plots verifying MAFG knockdown in the transferred compartment, corresponding to the representative plots in Figure 6G. (**C–H**) Supplementary Y-maze parameters: novel-arm zone occupancy (**C**; p < 0.0001), novel-arm entry percentage (**D**), latency to enter the novel arm (**E**), total arm entries (**F**), distance moved (**G**; p = 0.0288), and velocity (**H**). (**I**) Representative Y-maze occupancy heatmaps (sh-NC vs sh-MAFG). (**J–L**) Supplementary Morris water maze parameters: number of platform crossings (**J**), swim velocity (**K**; p = 0.0118), and distance to the target zone (**L**; p = 0.0002). (**M**) Representative Morris water maze occupancy heatmaps. Data are presented as mean ± SEM; statistical significance was assessed by unpaired Student’s t-test. NS, not significant.

**FIGURE | S8** Supplementary amyloid pathology in the adoptive-transfer cohort. (**A**) Representative amyloid plaque staining (blue; individual plaques circled in red) in sh-NC and sh-MAFG recipient brains. (**B**) Quantification of average microglial branch length (NS). (**C**) Brain Aβ1–42 levels measured by ELISA in cortex and hippocampus, showing a significant reduction with MAFG silencing (p < 0.0001 for both regions). Data are presented as mean ± SEM; statistical significance was assessed by unpaired Student’s t-test. NS, not significant.

**FIGURE | S9** Age and sex adjustment does not alter the MAFG-centred signature. (**A**) Donor age across disease states; age did not differ significantly between the healthy control, Alzheimer's disease and sepsis cohorts (Kruskal–Wallis p = 0.456), and pairwise equivalence was confirmed by two one-sided tests (Table S1B). (**B**) Genome-wide comparison of log2 fold-changes from the age-naive (~ disease state) and age + sex-adjusted models across all terminal subpopulations and disease contrasts, with the 13 core transcription factors highlighted (r = 0.98). (**C**) The same comparison restricted to well-expressed genes (baseMean ≥ 10), which removes low-count genes with unstable fold-change estimates (Pearson r = 0.868, Spearman rho = 0.977, sign agreement 94.8%); the signature genes NFIL3 and RGS10 are highlighted. (**D**) Sensitivity analysis in an age-matched sub-cohort restricted to the age window supported by all three disease states (47.5–87.0 years), showing that log2 fold-changes for the four signature genes are reproduced relative to the full age-adjusted cohort. (**E**) Effect sizes (log2 fold-change with 95% confidence interval) of the four signature genes (MAFG, NFIL3, RGS10, FOXO3) before (grey) and after (blue) age + sex adjustment, shown for each terminal subpopulation (common myeloid progenitor_c3, DC_c9-FCER1A, Platelet_c0, Platelet_c1) and each disease contrast (AD vs HC, sepsis vs AD, sepsis vs HC); adjustment did not change the direction or significance of any signature gene. (**F**) Overlap of differentially expressed gene lists (adjusted p < 0.05, |log2FC| > 0.25) called by the age-naive and age-adjusted models, per subpopulation and contrast. (**G**) MAFG pseudo-bulk expression (log2 CPM) plotted against donor age within each disease group, shown separately for the four terminal subpopulations; MAFG did not track age within any group, indicating that age does not explain the between-group difference. Dashed lines in (B–D) indicate the identity line. ns, not significant; *p < 0.05, **p < 0.01, ***p < 0.001, ****p < 0.0001.

**FIGURE | S10** Physiological recovery precedes behavioral testing in the composite APP/PS1+CLP model. (**A**) Kaplan–Meier survival after surgery for APP/PS1-Sham (n = 20) and APP/PS1-CLP mice (n = 38); mortality was confined to the first 18 days and no further deaths occurred thereafter (log-rank p = 0.007). (**B**) Body weight expressed as a percentage of the pre-surgical baseline, (**C**) daily food intake (g/mouse/day), (**D**) murine sepsis score and (**E**) home-cage activity (percentage of the sham mean), from 3 days before surgery to day 30. APP/PS1-CLP mice showed the expected acute sickness response, with maximal weight loss, anorexia, peak sepsis score and suppressed home-cage activity between days 1 and 3, followed by progressive recovery; by day 30 all four parameters had returned to the APP/PS1-Sham range. Faint lines show individual animals and bold lines the group mean ± SEM. p values are for the group × time interaction by two-way repeated-measures ANOVA. Behavioral testing was performed on day 30, after the entire period of acute mortality and after full resolution of sickness behavior, so that transient systemic illness could not confound the cognitive read-outs.
