# Supplementary material for: MAFG as a Redox‐Sensitive Node Linking Peripheral Immune Reprogramming to Cognitive Risk in the Sepsis‐to‐Alzheimer's Disease Continuum: Single‐Cell Dissection and Adoptive‐Transfer Validation
Source: CNS Neurosci Ther. 2026 Oct 13;32(10):e71201. doi: 10.1002/cns.71201 (PMC13659262; doi:10.1002/cns.71201)
Supplement: Supplementary file 2 [file CNS-32-e71201-s002.docx]

**Supplementary Methods**

Detailed protocols corresponding to the condensed Materials and Methods in the main text. Reagents, antibodies, shRNA constructs and primer sequences are listed in Table S2; dataset details are listed in Table S1.

**S1 | Single-cell data processing and clustering**

Raw expression matrices were processed using Seurat [1]. Genes expressed in fewer than three cells, and cells with gene counts outside 200–3000 or > 10% mitochondrial transcripts, were excluded. Filtered data were normalized and scaled, and the top 2000 variable features were selected. Batch effects were corrected by Harmony [2], followed by PCA-based dimensionality reduction and UMAP visualization. Unsupervised clustering was performed with "FindNeighbors" and "FindClusters", and clusters were annotated based on canonical marker genes. Cluster-specific marker genes and condition-specific differentially expressed genes (DEGs) were identified by Wilcoxon rank-sum test with Bonferroni correction (|log2FC| > 1, FDR < 0.05). To mitigate the influence of inter-individual variability and pseudo-replication inherent to cell-level testing, key disease-state comparisons were additionally confirmed using a patient-level pseudo-bulk approach, in which counts were aggregated per individual prior to differential expression analysis.

**S2 | Compositional and tissue-preference analysis**

Cell-type composition was compared across the HC, AD and sepsis cohorts at both the cluster and major-lineage levels. Disease preference was quantified using the STARTRAC-dist index [3] and the ratio of observed to expected cell numbers (Ro/e), the latter derived from a chi-square test, together with odds ratio (OR) analysis; an OR > 1.5 or Ro/e > 1 was taken to indicate preferential enrichment of a cluster or lineage in a given disease state, and enrichment preferences were annotated as positive, neutral or negative. Cluster-level abundance differences among the three cohorts were assessed by unpaired comparison and visualized as stacked composition plots and preference heatmaps.

**S3 | Trajectory inference and gene regulatory network analysis**

Pseudotime trajectories of peripheral immune subpopulations were constructed using Monocle2 [4] based on highly variable genes from the "differentialGeneTest" function, with dynamic gene expression modeled by a generalized additive model. Because the integrated datasets derive from independent sepsis and AD individuals rather than a single longitudinally sampled cohort, the resulting pseudotime ordering was interpreted as a cross-sectional, data-driven approximation of cell-state relationships rather than an authentic temporal progression.

Gene regulatory networks (regulons) were inferred using the SCENIC pipeline [5]. In brief, candidate transcription factor (TF)–target co-expression modules were identified by GENIE3 [6]; modules were refined by RcisTarget [5] cis-regulatory motif enrichment, retaining only targets supported by a significant motif near the transcription start site (normalized enrichment score > 3.0) to define high-confidence regulons; and regulon activity was scored per cell by AUCell [5]. Highly variable regulons were defined as those whose AUCell activity varied most across disease states (top regulons by variance), and the AUCell assignment threshold was determined automatically from the bimodal distribution of each regulon's activity histogram as implemented in AUCell. DEGs used for network construction applied the same |log2FC| > 1 and adjusted p < 0.05 thresholds described above.

**S4 | Functional enrichment and gene set enrichment analysis**

DEGs were annotated using "clusterProfiler" [7]; Gene Ontology (GO) and KEGG enrichment analyses were performed at q < 0.01 and p < 0.05. Pathway-level dysregulation associated with core biomarkers was assessed by GSEA [8] against MSigDB [9] gene sets. In addition, single-sample GSEA (ssGSEA) was performed with the GSVA R package [10] to score, per sample, the pathway activity associated with individual candidate biomarkers in the peripheral-blood (GSE65682) and brain-tissue (GSE135838) cohorts.

**S5 | Animal husbandry, sepsis model and sampling**

Male C57BL/6 mice (8–9 weeks old, 18–20 g) were acclimatized for one week prior to experiments. Male wild-type C57BL/6 mice (8–9 weeks old, 18–20 g) and age-matched male APP/PS1 double-transgenic mice B6-Tg(PrP-hAPP/hPS1) (APP/PS1 mice) were purchased from a commercial supplier (Beijing HFK Bio-Technology Co., Ltd) and acclimatized for one week under specific-pathogen-free conditions.

Polymicrobial sepsis was induced by cecal ligation and puncture (CLP) under isoflurane anesthesia [11]; the distal one-third of the cecum was ligated and punctured once with a 22-gauge needle, and the abdomen was closed. Sham-operated mice underwent identical procedures without ligation or puncture. At pre-defined time points (8 h to 30 days post-surgery), mice were euthanized by intraperitoneal overdose of sodium pentobarbital (150 mg/kg), and peripheral blood and bone marrow were harvested. Blood was centrifuged (3000 rpm, 10 min, 4 °C) to isolate serum and PBMCs for flow cytometric and molecular analyses.

**S6 | Western blot and quantitative RT-PCR**

Total protein from PBMCs was extracted with RIPA buffer, separated by 10% SDS-PAGE, transferred to PVDF membranes, blocked, and incubated with primary and HRP-conjugated secondary antibodies. β-actin served as the loading control. Bands were visualized by ECL substrate and quantified with ImageJ. Total RNA was extracted by the Trizol method, reverse-transcribed into cDNA, and quantified by SYBR Green-based qPCR; relative expression was calculated by the 2^−ΔΔCt^ method with 18S rRNA as the internal control.

**S7 | Flow cytometry and oxidative stress assays**

Multicolor flow cytometry was performed on murine peripheral blood and bone marrow. After erythrocyte lysis and PBMC isolation, dendritic cells (DCs) were pre-enriched with CD11c magnetic beads prior to staining for common myeloid progenitors (CMPs), DCs and platelets. Intracellular MAFG was detected using the Foxp3/Transcription Factor Staining Buffer Set. Data were acquired on a BD LSRFortessa flow cytometer and analyzed with FlowJo. The complete gating strategy for BM-CMP, BD-CMP, BD-DC and BD-Platelet is provided in Figure S5B.

Serum malondialdehyde (MDA) and total antioxidant capacity (T-AOC) were quantified using commercial colorimetric kits according to the manufacturers' instructions, with optical density read on a multimode microplate reader.

**S8 | Behavioral testing**

Cognitive and locomotor function was assessed under dim, low-noise conditions by an investigator blinded to genotype and treatment, with the apparatus cleaned with 70% ethanol between animals and all trials recorded and scored by an automated video-tracking system.

**Novel object recognition (NOR).** Performed in an open-field arena: after habituation, mice explored two identical objects during a familiarization trial and, after a retention interval, were re-exposed to one familiar and one novel object. Exploration time for each object was used to compute the recognition index [RI = t_novel / (t_novel + t_familiar) × 100%] and the discrimination index [DI = (t_novel − t_familiar) / (t_novel + t_familiar)], while total distance moved and mean velocity were recorded to exclude locomotor confounds.

**Y-maze.** Spatial working memory was evaluated in a symmetrical three-arm Y-maze. Spontaneous alternation was scored as the percentage of consecutive entries into three different arms [alternation% = actual alternations / (total entries − 2) × 100%]. In the novel-arm variant, one arm was blocked during training and opened at test, with novel-arm entries, dwell time and latency quantified.

**Morris water maze (MWM).** During acquisition, mice were trained over consecutive days to locate a hidden platform using distal spatial cues (escape latency recorded), followed by a probe trial with the platform removed, during which time in the target quadrant, number of platform crossings, swim velocity, and distance/latency to the target zone were measured. Occupancy heatmaps were generated from the tracking data.

**S9 | Brain tissue processing, immunostaining and microglial morphometry**

At the experimental endpoint, mice were deeply anesthetized and transcardially perfused with ice-cold PBS followed by 4% paraformaldehyde; brains were post-fixed, cryoprotected in sucrose, and cut into coronal sections. Amyloid plaques were detected by 4G8 immunostaining and, in parallel, by Methoxy-X04 histochemistry with DAPI nuclear counterstaining, and microglia were labeled by Iba1 immunofluorescence (Alexa Fluor 488). Images were acquired by fluorescence/confocal microscopy under identical settings across groups.

Amyloid burden—plaque load (area fraction), plaque density, plaque count per field and mean plaque area—and Iba1⁺ parameters (cell density and area fraction) were quantified in the cortex and hippocampus using ImageJ/FIJI [12] by a blinded investigator. Microglial morphology was reconstructed by skeletonization (AnalyzeSkeleton [13]) and Sholl analysis [14], yielding soma area, territory (domain) area, morphology index (territory area / soma area), circularity, solidity, total process length, numbers of branches, junctions (branch points) and end-points, and average and maximum branch lengths. Brain Aβ1–42 was quantified by sandwich ELISA in cortical and hippocampal homogenates according to the manufacturer's instructions.

**S10 | Isolation, lentiviral MAFG knockdown and functional profiling of peripheral immune cells**

Primary peripheral immune cells were isolated from blood by density-gradient centrifugation followed by fluorescence-activated cell sorting. For loss-of-function studies, MAFG was silenced by lentiviral delivery of a MAFG-targeting short hairpin RNA (sh-MAFG), with a non-targeting shRNA (sh-NC) as control. Lentivirus was packaged in-house by co-transfecting the sh-MAFG (or sh-NC) shRNA-expressing transfer plasmid together with the packaging plasmids of a commercial lentiviral packaging system (GeneChem) into 293T cells; culture supernatants were harvested, and viral particles were concentrated and purified by ultracentrifugation prior to transduction of the sorted peripheral immune cells. Knockdown efficiency was confirmed by RT-qPCR and by flow cytometry for intracellular MAFG.

To assess Aβ clearance, cells were incubated with fluorescently labeled Aβ; extracellular (non-internalized) fluorescence was then quenched with trypan blue so that flow cytometry quantified only internalized Aβ, and the proportion of Aβ⁺ (phagocytic) cells was determined, with untreated cells as a negative gating control. To assess inflammatory responses, cells were left unstimulated or stimulated with lipopolysaccharide (LPS), and secreted IL-1β, IL-6 and TNF-α were measured by ELISA; in parallel, the mRNA levels of Aβ-clearance/scavenger receptor genes (CD33, TLR2, MSR1 and SYK) and pro-inflammatory genes were quantified by RT-qPCR.

**S11 | Adoptive cell transfer**

For adoptive-transfer experiments, 6-month-old APP/PS1 recipient mice underwent a baseline Y-maze test and then received serial tail-vein transfers of sh-NC or sh-MAFG peripheral cells (four transfers over the treatment period; experimental timeline in Figure 6F). Persistence of MAFG knockdown in the transferred compartment was verified in recipient blood by flow cytometry (MAFG mean and median fluorescence intensity and the MAFG⁺ fraction of CD45⁺ cells). Recipients subsequently underwent Y-maze and Morris water maze testing, after which they were transcardially perfused for neuropathological and biochemical analysis.

**References**

1.	Y. Hao, S. Hao, E. Andersen-Nissen, et al., "Integrated analysis of multimodal single-cell data," *Cell* 184 (2021): 3573-3587.e29.

2.	I. Korsunsky, N. Millard, J. Fan, et al., "Fast, sensitive and accurate integration of single-cell data with Harmony," *Nat Methods* 16 (2019): 1289-1296.

3.	L. Zhang, X. Yu, L. Zheng, et al., "Lineage tracking reveals dynamic relationships of T cells in colorectal cancer," *Nature* 564 (2018): 268-272.

4.	X. Qiu, Q. Mao, Y. Tang, et al., "Reversed graph embedding resolves complex single-cell trajectories," *Nat Methods* 14 (2017): 979-982.

5.	S. Aibar, C.B. González-Blas, T. Moerman, et al., "SCENIC: single-cell regulatory network inference and clustering," *Nat Methods* 14 (2017): 1083-1086.

6.	V.A. Huynh-Thu, A. Irrthum, L. Wehenkel, et al., "Inferring regulatory networks from expression data using tree-based methods," *PLoS One* 5 (2010).

7.	T. Wu, E. Hu, S. Xu, et al., "clusterProfiler 4.0: A universal enrichment tool for interpreting omics data," *Innovation (Camb)* 2 (2021): 100141.

8.	A. Subramanian, P. Tamayo, V.K. Mootha, et al., "Gene set enrichment analysis: a knowledge-based approach for interpreting genome-wide expression profiles," *Proc Natl Acad Sci U S A* 102 (2005): 15545-50.

9.	A. Liberzon, C. Birger, H. Thorvaldsdóttir, et al., "The Molecular Signatures Database (MSigDB) hallmark gene set collection," *Cell Syst* 1 (2015): 417-425.

10.	S. Hänzelmann, R. Castelo, and J. Guinney, "GSVA: gene set variation analysis for microarray and RNA-seq data," *BMC Bioinformatics* 14 (2013): 7.

11.	D. Rittirsch, M.S. Huber-Lang, M.A. Flierl, et al., "Immunodesign of experimental sepsis by cecal ligation and puncture," *Nat Protoc* 4 (2009): 31-6.

12.	J. Schindelin, I. Arganda-Carreras, E. Frise, et al., "Fiji: an open-source platform for biological-image analysis," *Nat Methods* 9 (2012): 676-82.

13.	I. Arganda-Carreras, R. Fernández-González, A. Muñoz-Barrutia, et al., "3D reconstruction of histological sections: Application to mammary gland tissue," *Microsc Res Tech* 73 (2010): 1019-29.

14.	D.A. Sholl, "Dendritic organization in the neurons of the visual and motor cortices of the cat," *J Anat* 87 (1953): 387-406.
